# Supplementary material for: Evaluation of animal and plant diversity suggests Greenland’s thaw hastens the biodiversity crisis
Source: Commun Biol. 2022 Sep 17;5:985. doi: 10.1038/s42003-022-03943-3 (PMC9482659; doi:10.1038/s42003-022-03943-3)
Supplement: Supplementary file 3 — Description of Additional Supplementary Files [file 42003_2022_3943_MOESM3_ESM.pdf]

Description of Additional Supplementary Files

File name: Supplementary Data 1-6

Description: Folder behind the analyses, tables and figures from our main text and Supplementary material.
