# Supplementary material for: Evaluation of animal and plant diversity suggests Greenland’s thaw hastens the biodiversity crisis
Source: Commun Biol. 2022 Sep 17;5:985. doi: 10.1038/s42003-022-03943-3 (PMC9482659; doi:10.1038/s42003-022-03943-3)
Supplement: Supplementary file 2 — Supplementary Information [file 42003_2022_3943_MOESM2_ESM.pdf]

Evaluation of animal and plant diversity suggests Greenland's thaw hastens the biodiversity crisis

Carolina Ureta, Santiago Ramírez-Barahona, Óscar Calderón-Bustamante, Pedro Cruz-Santiago, Carlos Gay-García, Didier Swingedouw, Dimitri DeFrance, Angela P. Cuervo-Robayo

Supplementary Information

- This file contains Supplementary tables 1 and Supplementary figures 1–35.
- Supplementary data files and R code are available at Zenodo with the identifier http://doi.org/10.5281/zenodo.4917258
- Data and code citation (version 1.0):

Carolina Ureta, Santiago Ramírez Barahona, Óscar Calderón-Bustamante, Pedro Cruz-Santiago, Carlos Gay-García, Didier Swingedouw, Angela P. Cuervo-Robayo (2021) *Evaluation of animal and plant diversity suggests Greenland's thaw hastens the biodiversity crisis* (Version v1.0) [Data set]. Zenodo. http://doi.org/10.5281/zenodo.4917258

Table of contents

Supplementary tables and figures *4*

Supplementary table 1 4

Supplementary Figure 1 5

Supplementary Figure 2 6

Supplementary Figure 3 7

Supplementary Figure 4 8

Supplementary Figure 5 9

Supplementary Figure 6 10

Supplementary Figure 7 11

Supplementary Figure 8 12

Supplementary Figure 9 13

Supplementary Figure 10 14

Supplementary Figure 11 15

Supplementary Figure 12 16

Supplementary Figure 13 17

Supplementary Figure 14 18

Supplementary Figure 15 19

Supplementary Figure 16 20

Supplementary Figure 17 21

Supplementary Figure 18 22

Supplementary Figure 19 23

Supplementary Figure 20 24

Supplementary Figure 21 25

Supplementary Figure 22 26

Supplementary Figure 23 27

Supplementary Figure 24 28

Supplementary Figure 25 29

Supplementary Figure 26 30

Supplementary Figure 27 31

Supplementary Figure 28 .... **32**
Supplementary Figure 29 .... **33**
Supplementary Figure 30 .... **34**
Supplementary Figure 31 .... **35**
Supplementary Figure 32 .... **36**
Supplementary Figure 34 .... **38**
Supplementary Figure 35 .... **39**

Supplementary tables and figures

Supplementary table 1

Distribution modelling uncertainty represented by the coefficient of variation (CV). The coefficient of variation reflects the degree of agreement/disagreement in predictions across the seven algorithms used. Smaller values represent greater consensus between algorithms.

**Group**	**Mean CV**
Amphibians	0.059
Birds	0.056
Mammals	0.055
Reptiles	0.053
Ferns	0.075
Flowering plants	0.078
Gymnosperms	0.079
Lycophytes	0.080

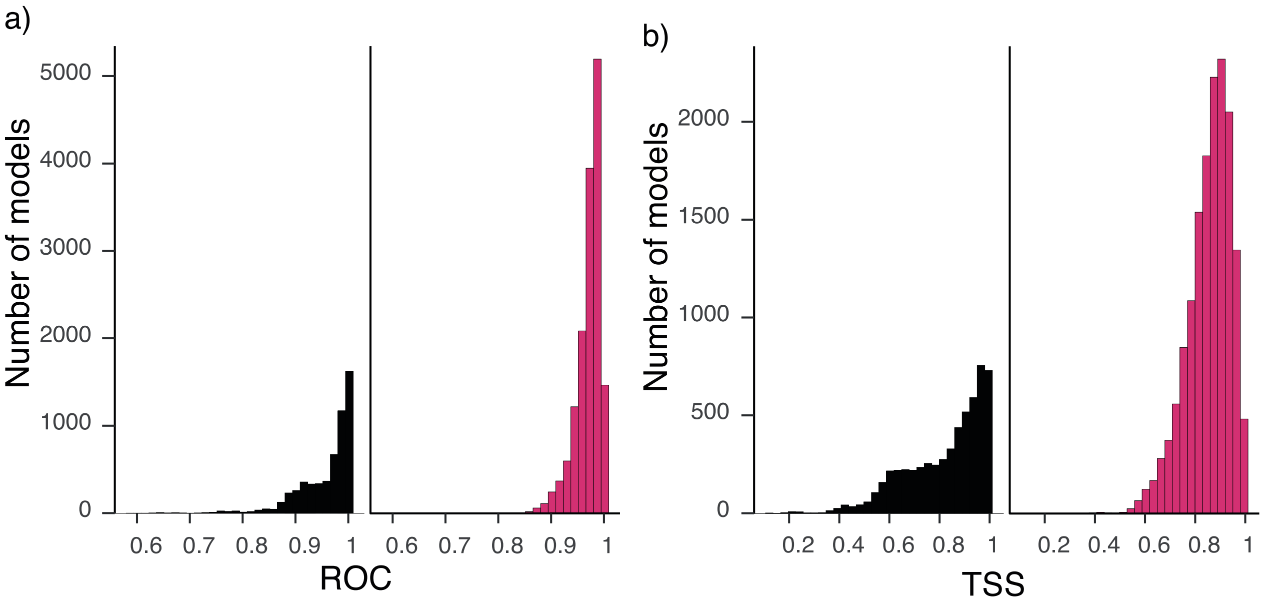

Supplementary Figure 1

Validation statistics for the ensemble species distribution models for tetrapods and vascular plants. a-b, histograms depicting the distribution of (a) ROC and (b) statistics for the 21,146 ensemble models for tetrapods (left panels) and vascular plants (right panels).

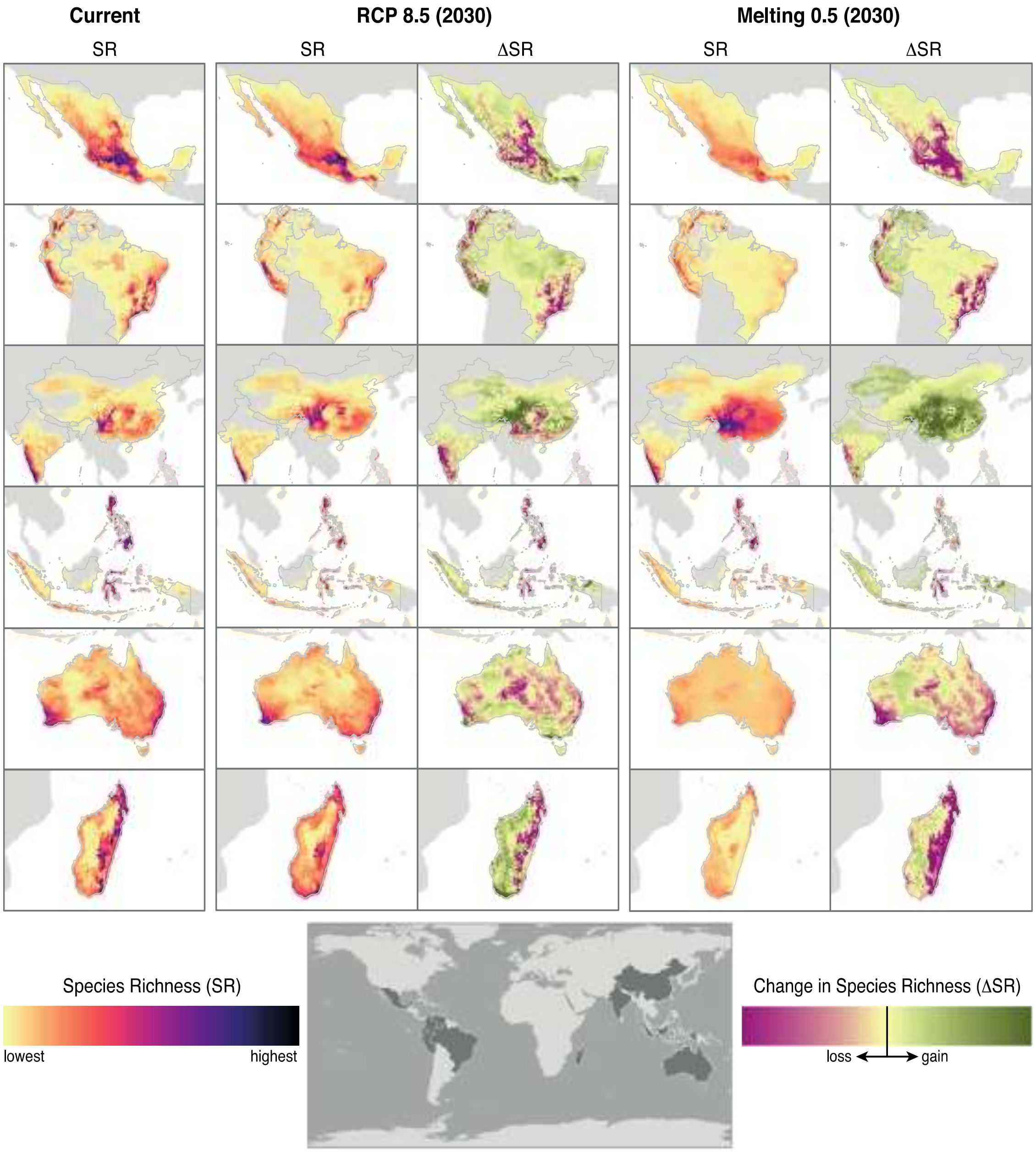

Supplementary Figure 2

Geographic patterns of present-day species richness and temporal changes in species ranges of vascular plants across twelve megadiverse countries. Estimates are based on species distribution models (SDMs) of vascular plants and tetrapods. Species richness (SR) was standardised to the range 0–1 within each country. Differences in species ranges (DSR) are shown for 2030–2060 under the RCP 8.5 and Melting 0.5 scenarios. For each grid-cell, the change in species ranges was estimated as the difference in the number of species that find suitable climatic conditions in a specific geographic area and scenario.

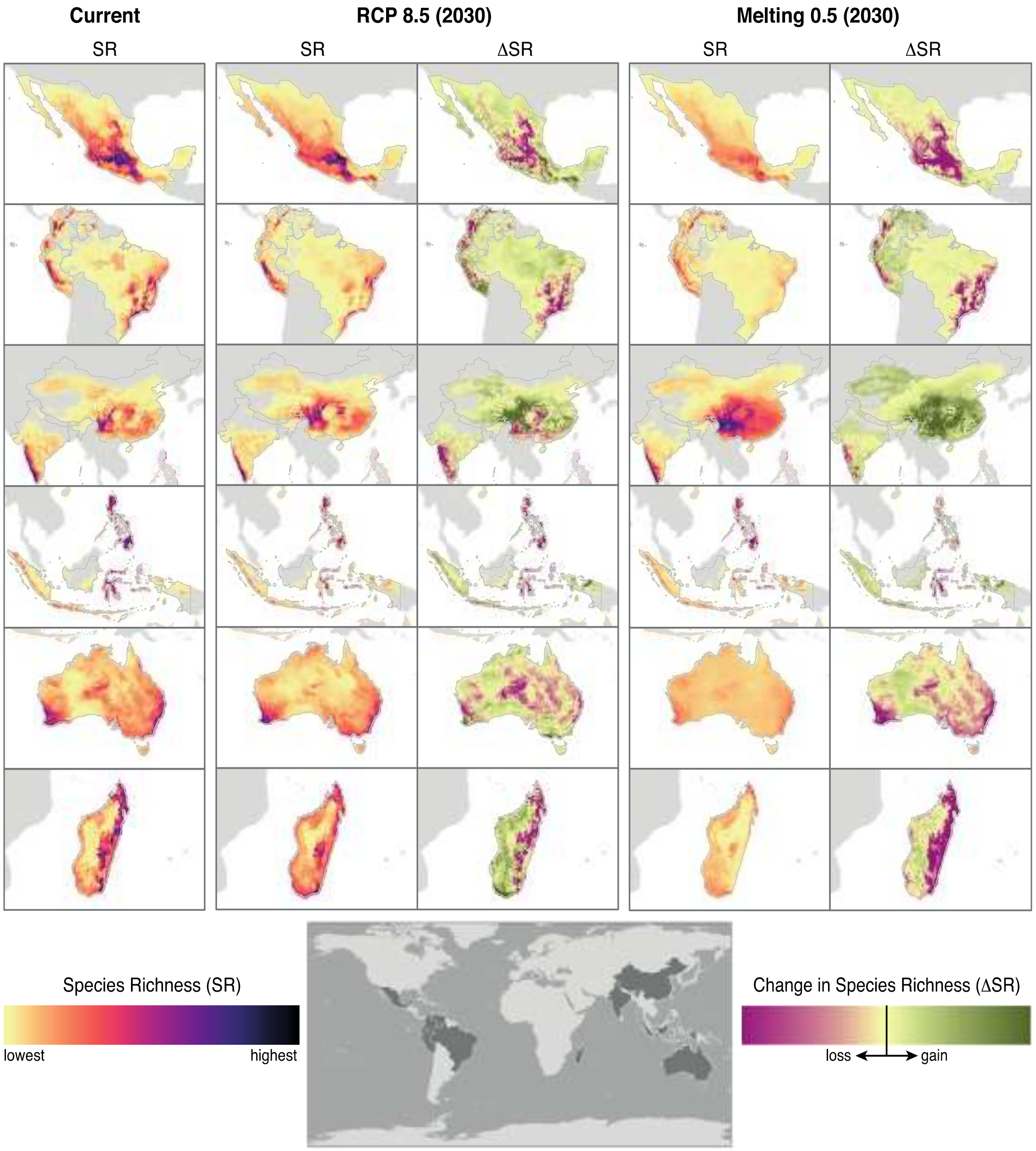

Supplementary Figure 3

Geographic patterns of present-day species richness and temporal changes in species richness of tetrapods across twelve megadiverse countries. Estimates are based on species distribution models (SDMs) of vascular plants and tetrapods. Species richness (SR) was standardised to the range 0–1 within each country. Differences in species richness (DSR) are shown for T1: 2030–2060 under the RCP 8.5 and Melting 0.5 scenarios. For each grid-cell, the change in species ranges was estimated as the difference in the number of species that find suitable climatic conditions in a specific geographic area and scenario.

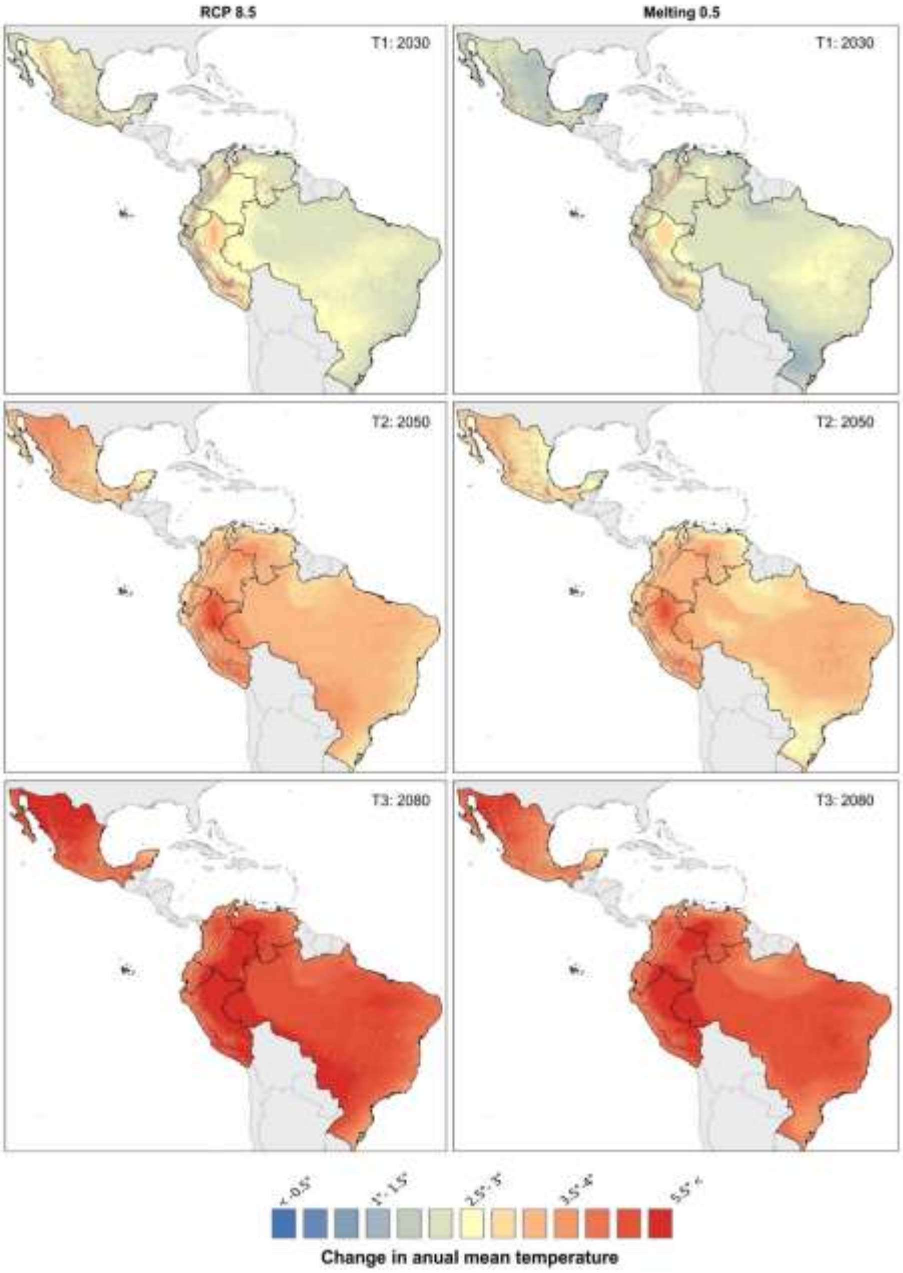

Supplementary Figure 4

Change in mean annual temperature (°C). Difference in annual mean temperature between present-day climatic conditions (1970–2000) and future scenarios for Mexico, Colombia, Ecuador, Peru, Brazil, and Venezuela at three consecutive time horizons: T1: 2030; T2: 2050; and T3: 2070.

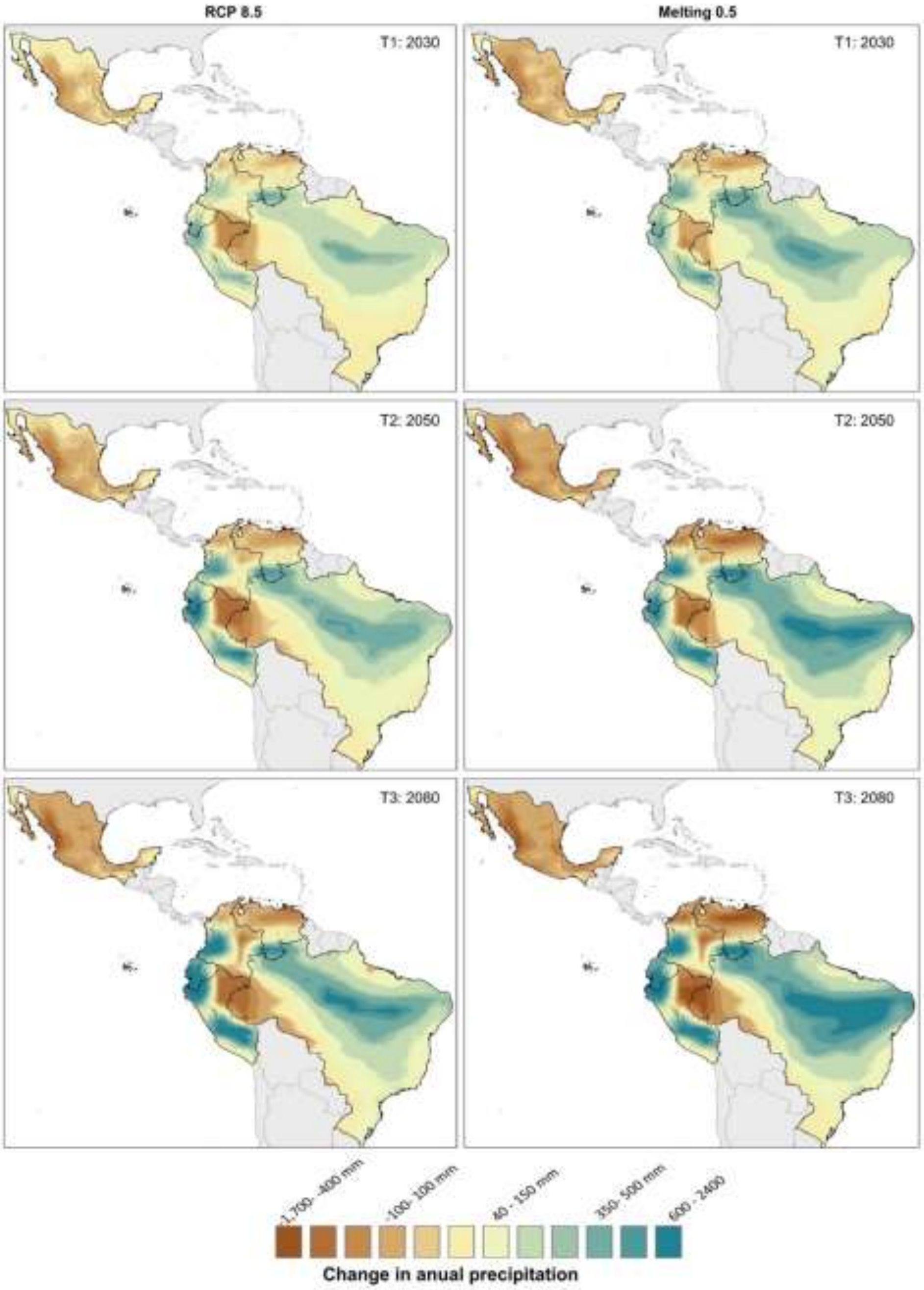

Supplementary Figure 5

Change in annual precipitation (mm). Difference in annual precipitation between present-day climatic conditions (1970–2000) and future scenarios for Mexico, Colombia, Ecuador, Peru, Brazil, and Venezuela at three consecutive time horizons: T1: 2030; T2: 2050; and T3: 2070.

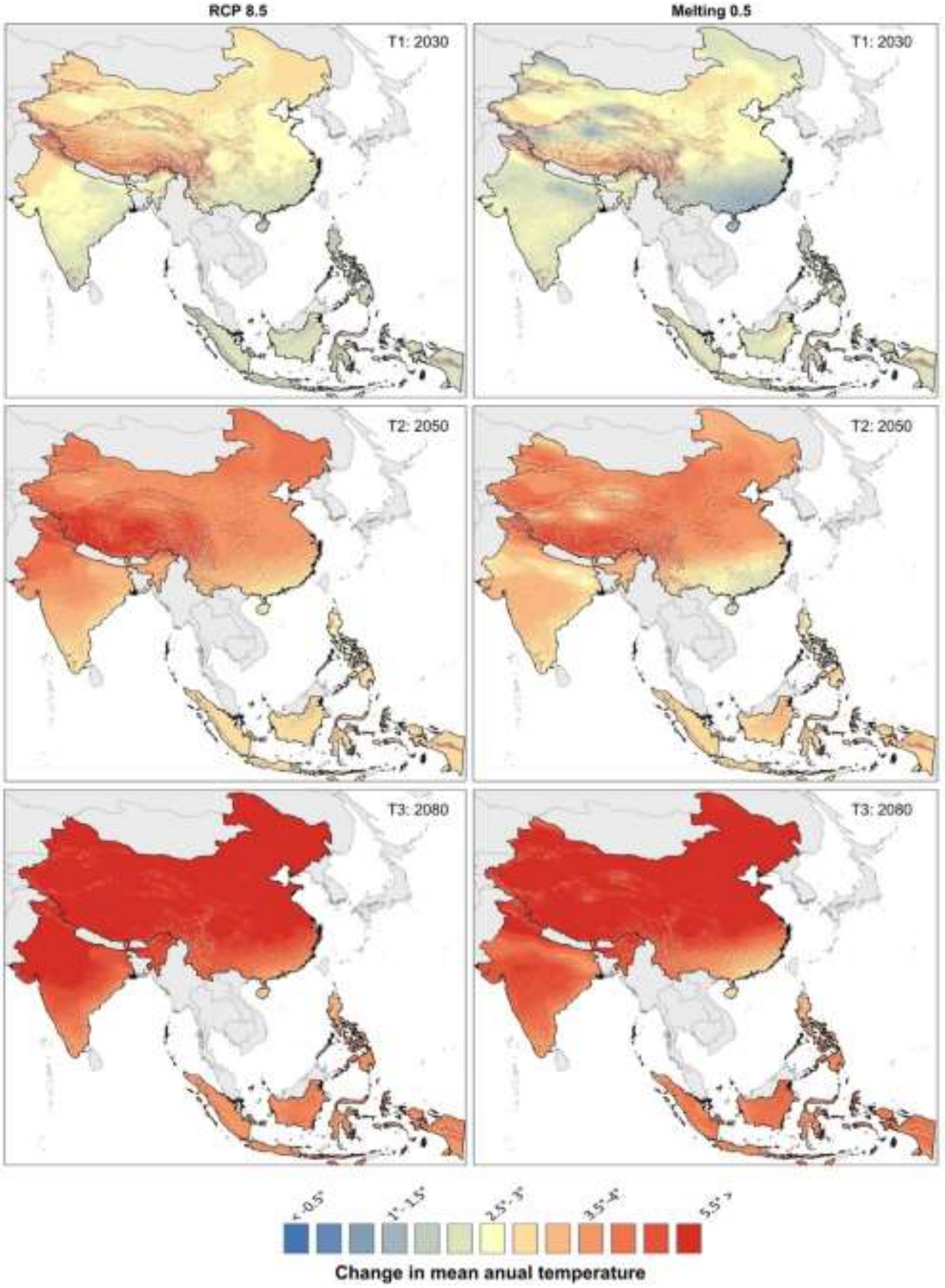

Supplementary Figure 6

Change in mean annual temperature (°C). Difference in annual mean temperature between present-day climatic conditions (1970–2000) and future scenarios for India, China, Philippines, and Indonesia at three consecutive time horizons: T1: 2030; T2: 2050; and T3: 2070.

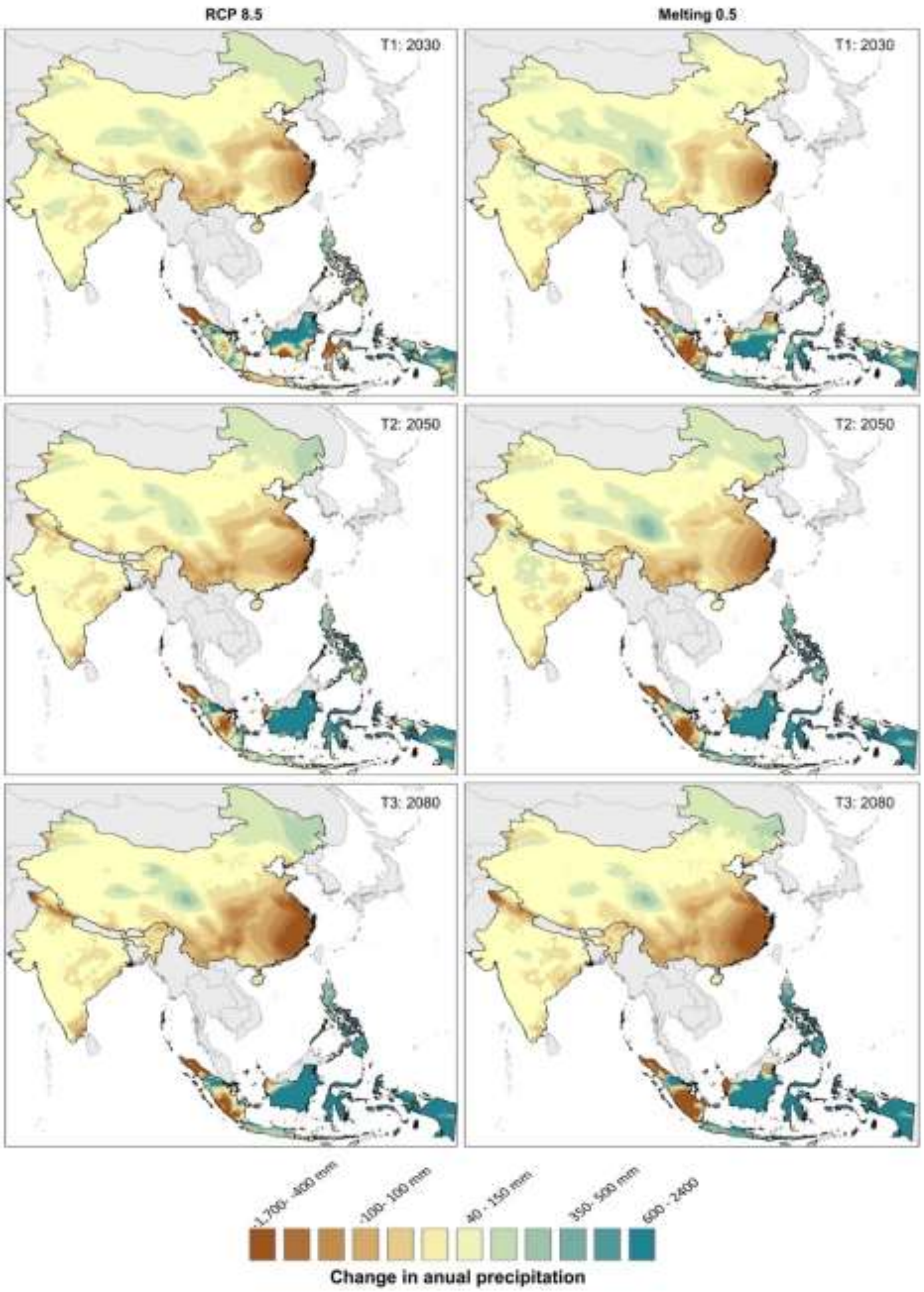

Supplementary Figure 7

Change in annual precipitation (mm). Difference in annual precipitation between present-day climatic conditions (1970–2000) and future scenarios for India, China, Philippines, and Indonesia at three consecutive time horizons: T1: 2030; T2: 2050; and T3: 2070.

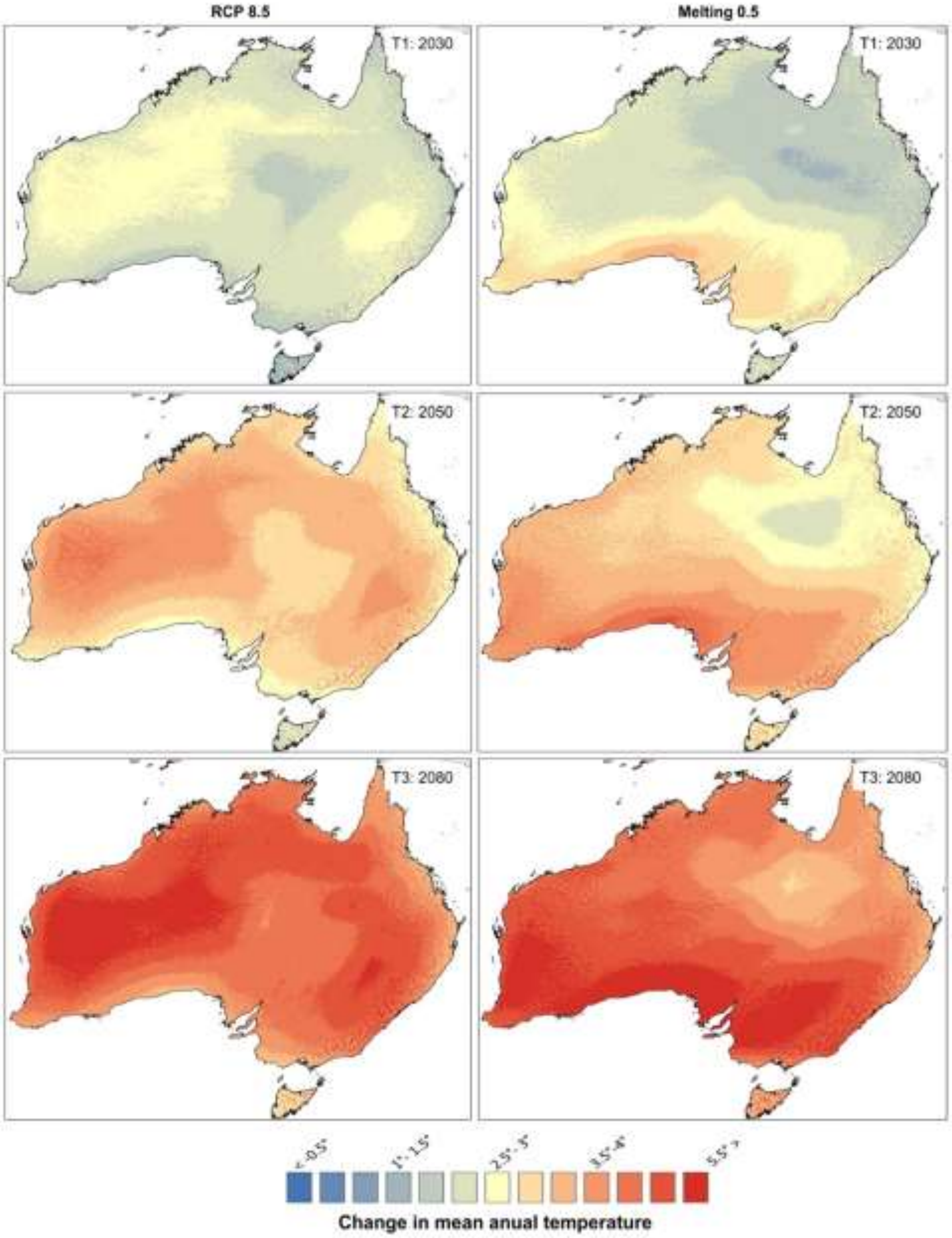

Supplementary Figure 8

Change in mean annual temperature (°C). Difference in mean annual temperature between present-day climatic conditions (1970–2000) and future scenarios for Australia at three consecutive time horizons: T1: 2030; T2: 2050; and T3: 2070.

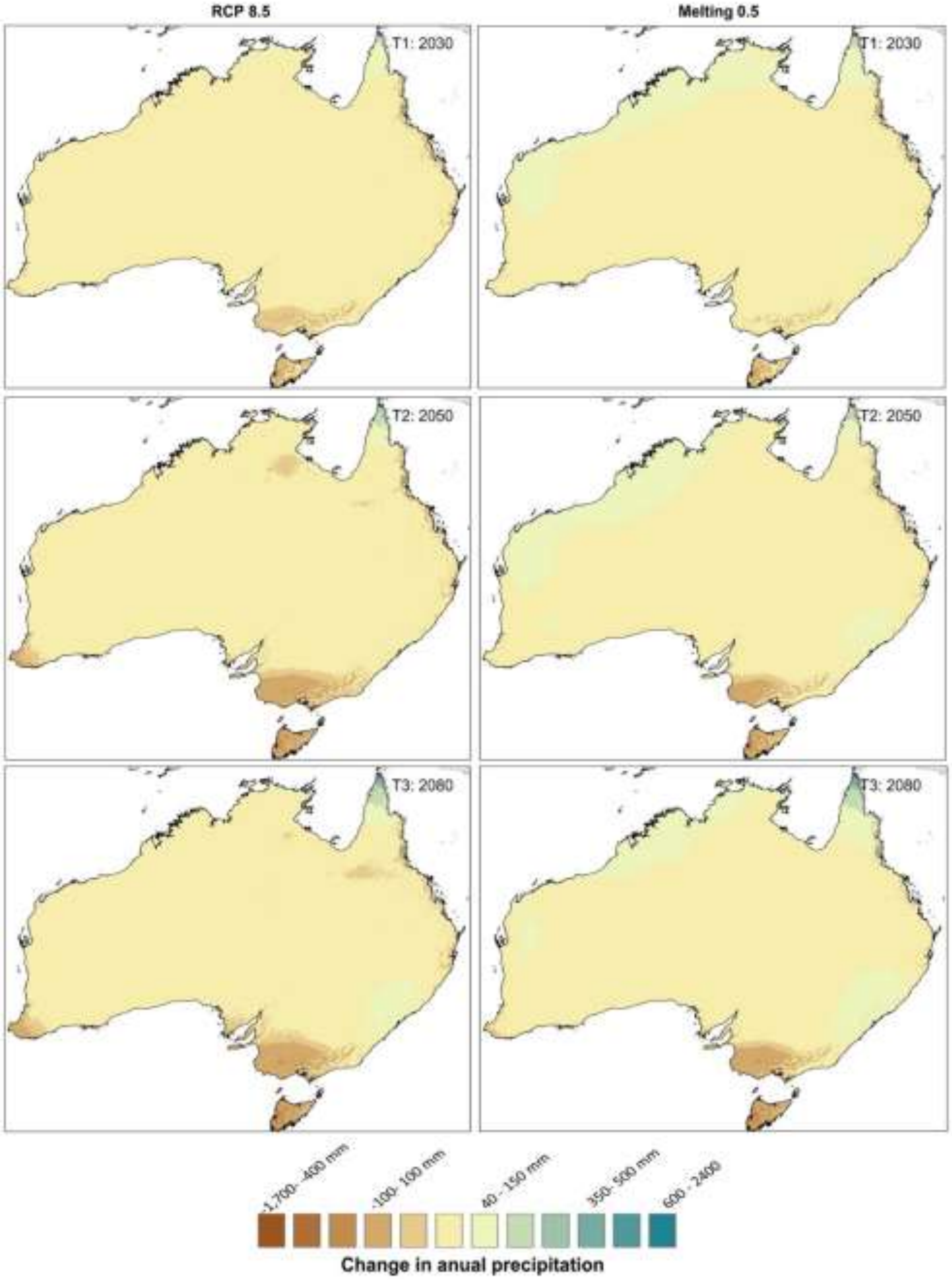

Supplementary Figure 9

Change in annual precipitation difference (mm). Difference in annual precipitation between present-day climatic conditions (1970–2000) and future scenarios for Australia at three consecutive time horizons: T1: 2030; T2: 2050; and T3: 2070.

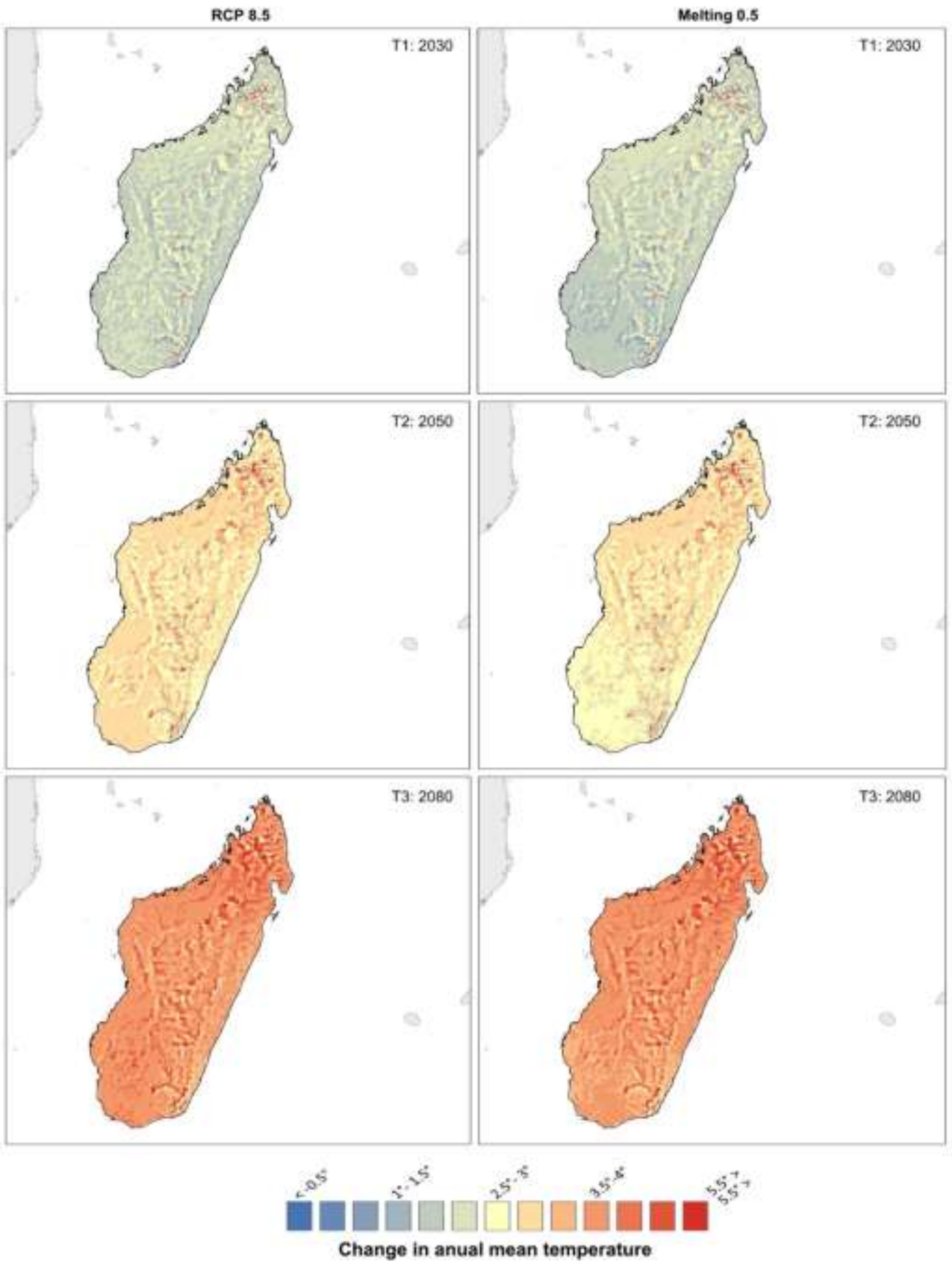

Supplementary Figure 10

Change in mean annual temperature (°C). Difference in annual mean temperature between present-day climatic conditions (1970–2000) and future scenarios for Madagascar at three consecutive time horizons: T1: 2030; T2: 2050; and T3: 2070.

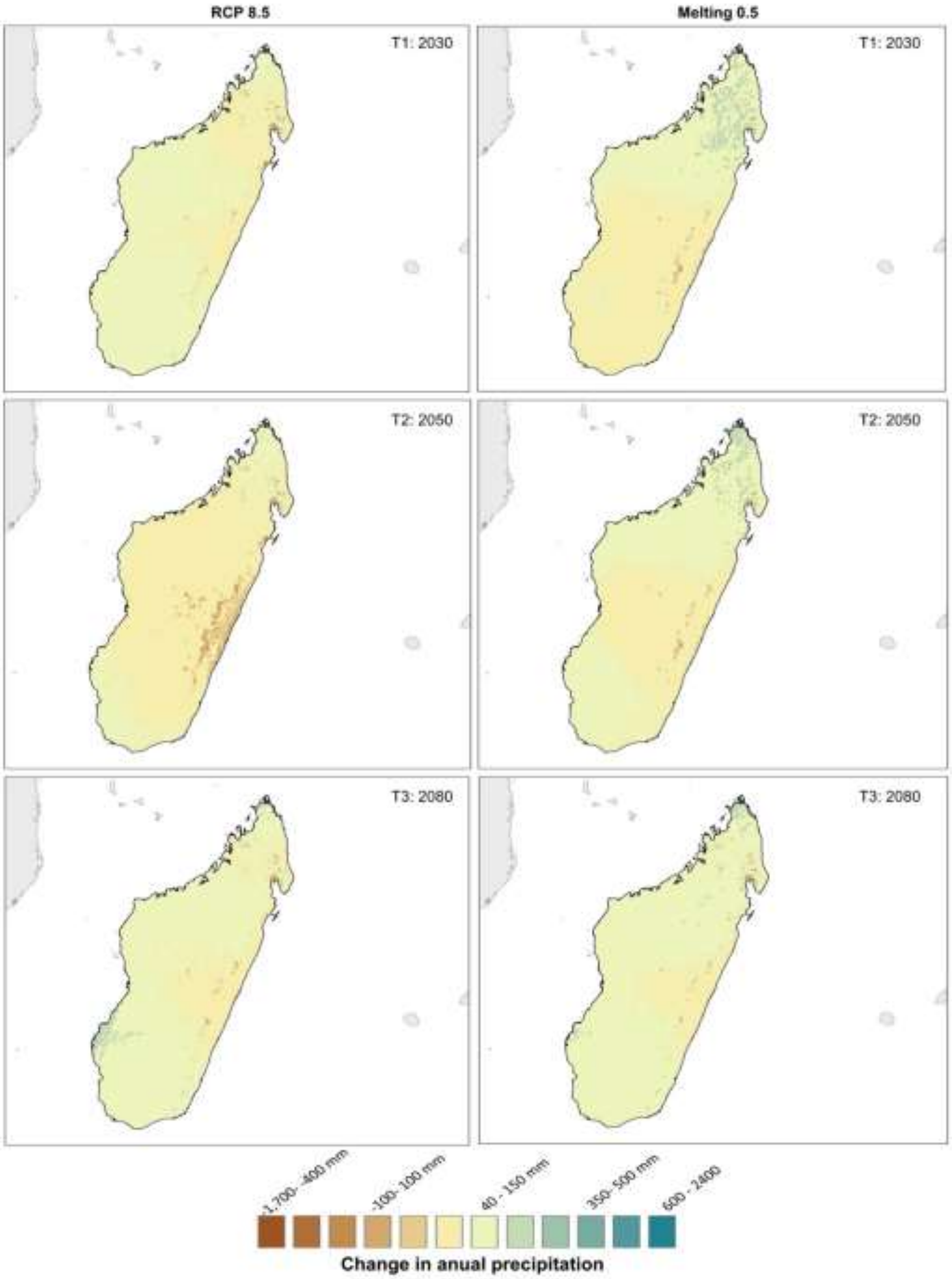

Supplementary Figure 11

Change in Annual precipitation difference (mm). Difference in annual precipitation between present-day climatic conditions (1970–2000) and future scenarios for Madagascar at three consecutive time horizons: T1: 2030; T2: 2050; and T3: 2070.

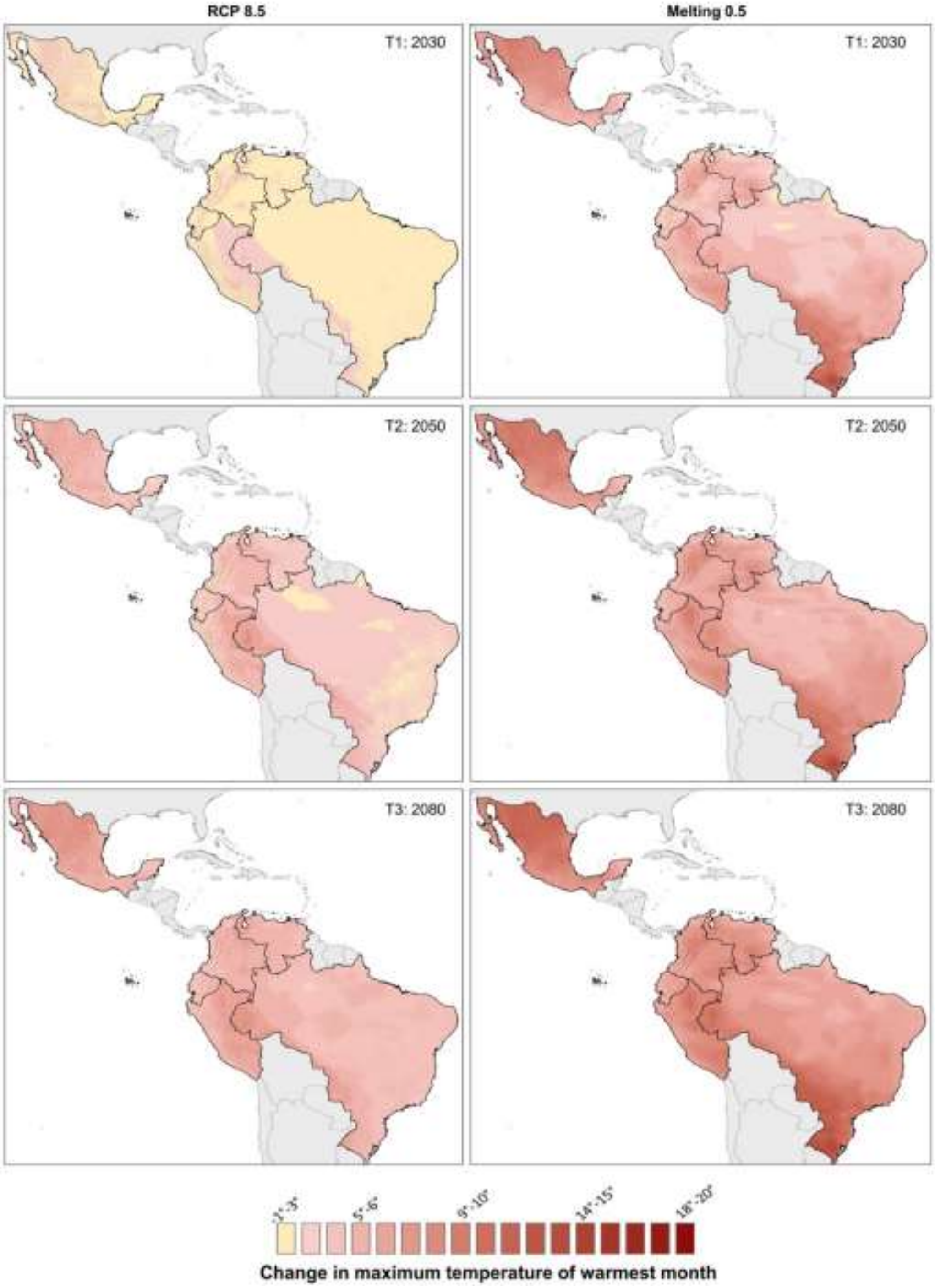

Supplementary Figure 12

Change in maximum temperature of the warmest month (°C). Difference in the maximum temperature of the warmest month between present-day climatic conditions (1970–2000) and future scenarios for Mexico, Colombia, Ecuador, Peru, Brazil and Venezuela at three consecutive time horizons: T1: 2030; T2: 2050; and T3: 2070.

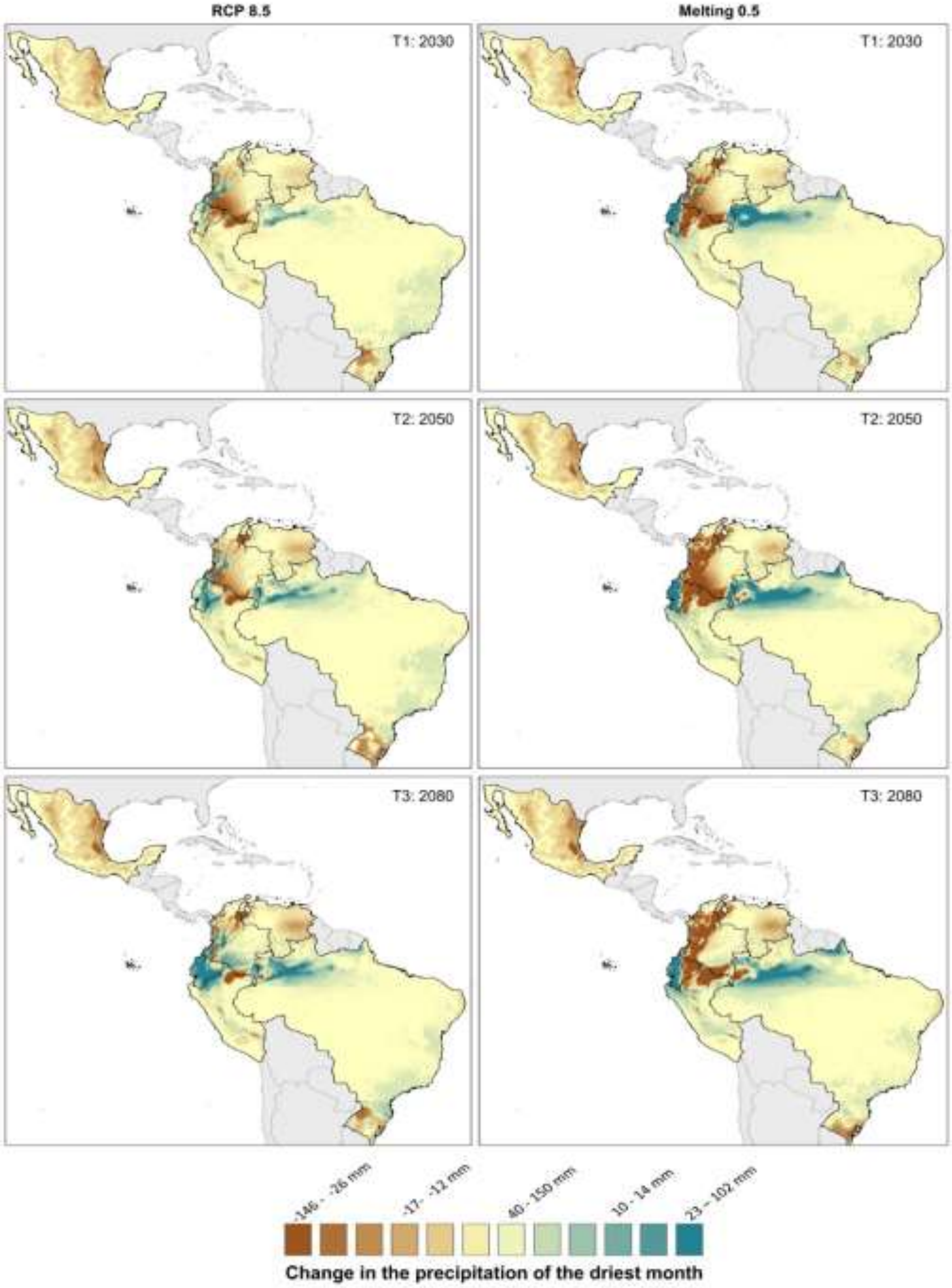

Supplementary Figure 13

Change in precipitation of the driest month (mm). Difference in precipitation of the driest month between present-day climatic conditions (1970–2000) and future scenarios for Mexico, Colombia, Ecuador, Peru, Brazil and Venezuela at three consecutive time horizons: T1: 2030; T2: 2050; and T3: 2070.

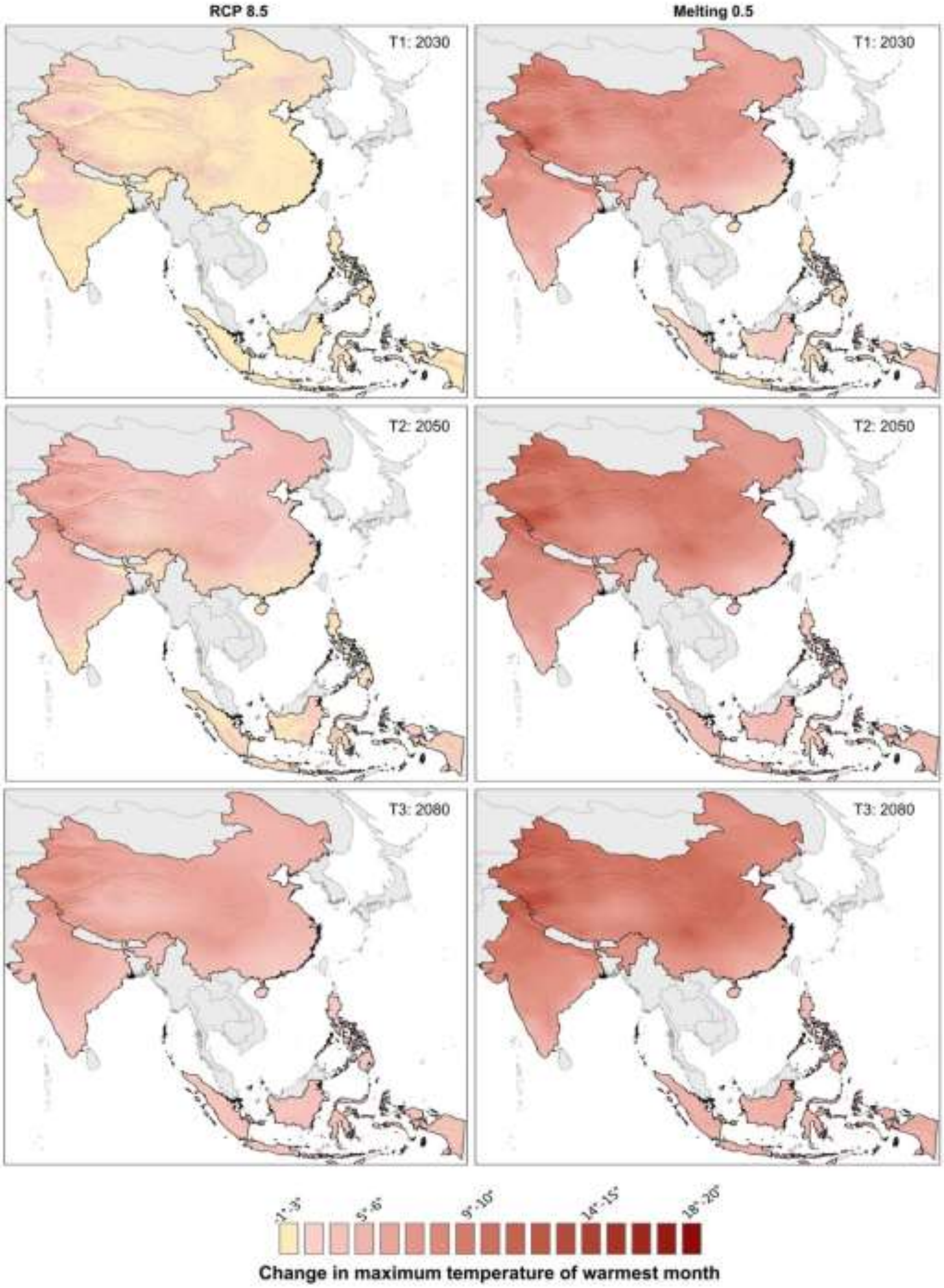

Supplementary Figure 14

Change in maximum temperature of the warmest month (°C). Difference in the maximum temperature of the warmest month between present-day climatic conditions (1970–2000) and future scenarios for India, China, Philippines, and Indonesia at three consecutive time horizons: T1: 2030; T2: 2050; and T3: 2070.

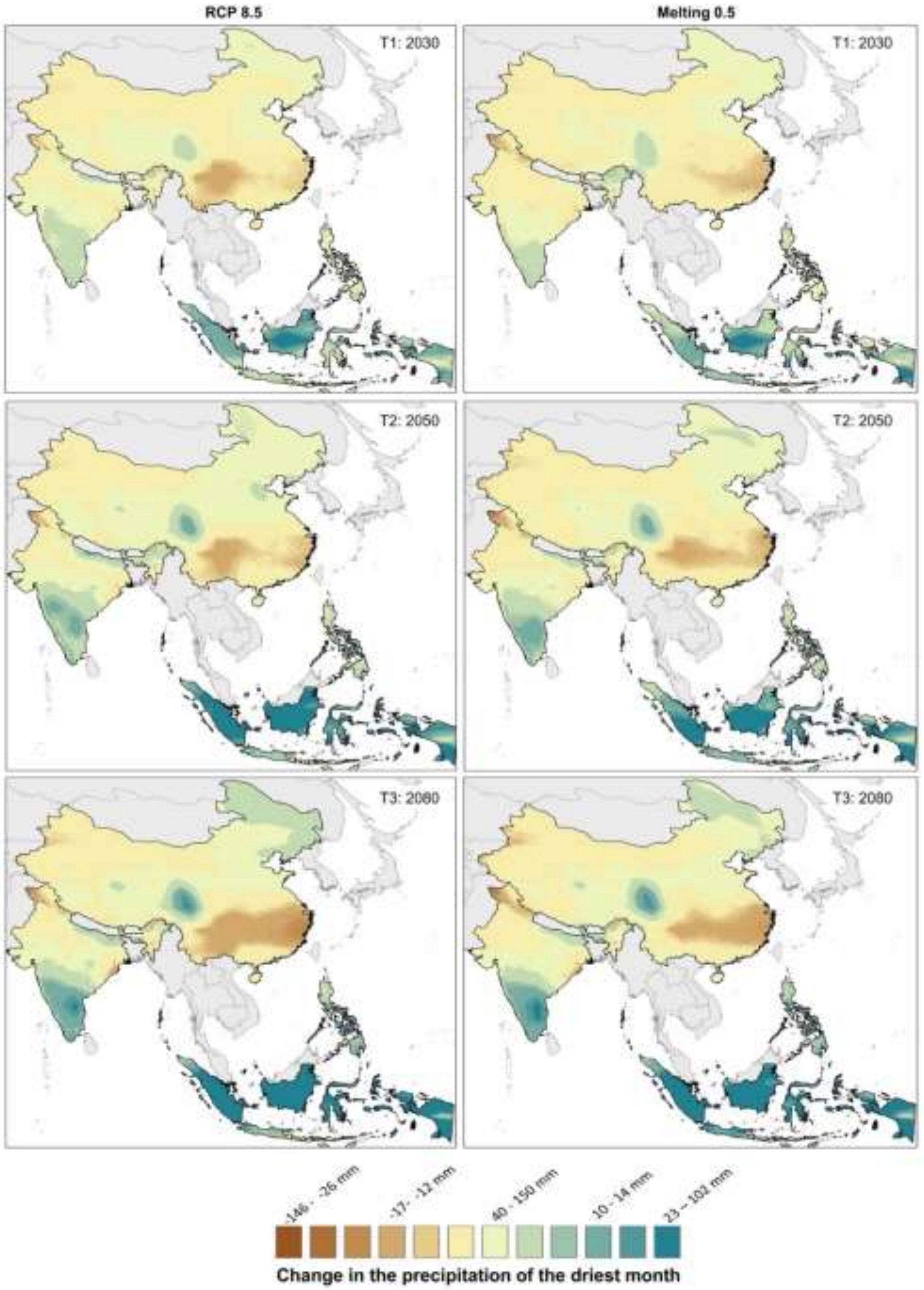

Supplementary Figure 15

Change in precipitation of the driest month (mm). Difference in precipitation of the driest month between present-day climatic conditions (1970–2000) and future scenarios for India, China, Philippines, and Indonesia at three consecutive time horizons: T1: 2030; T2: 2050; and T3: 2070.

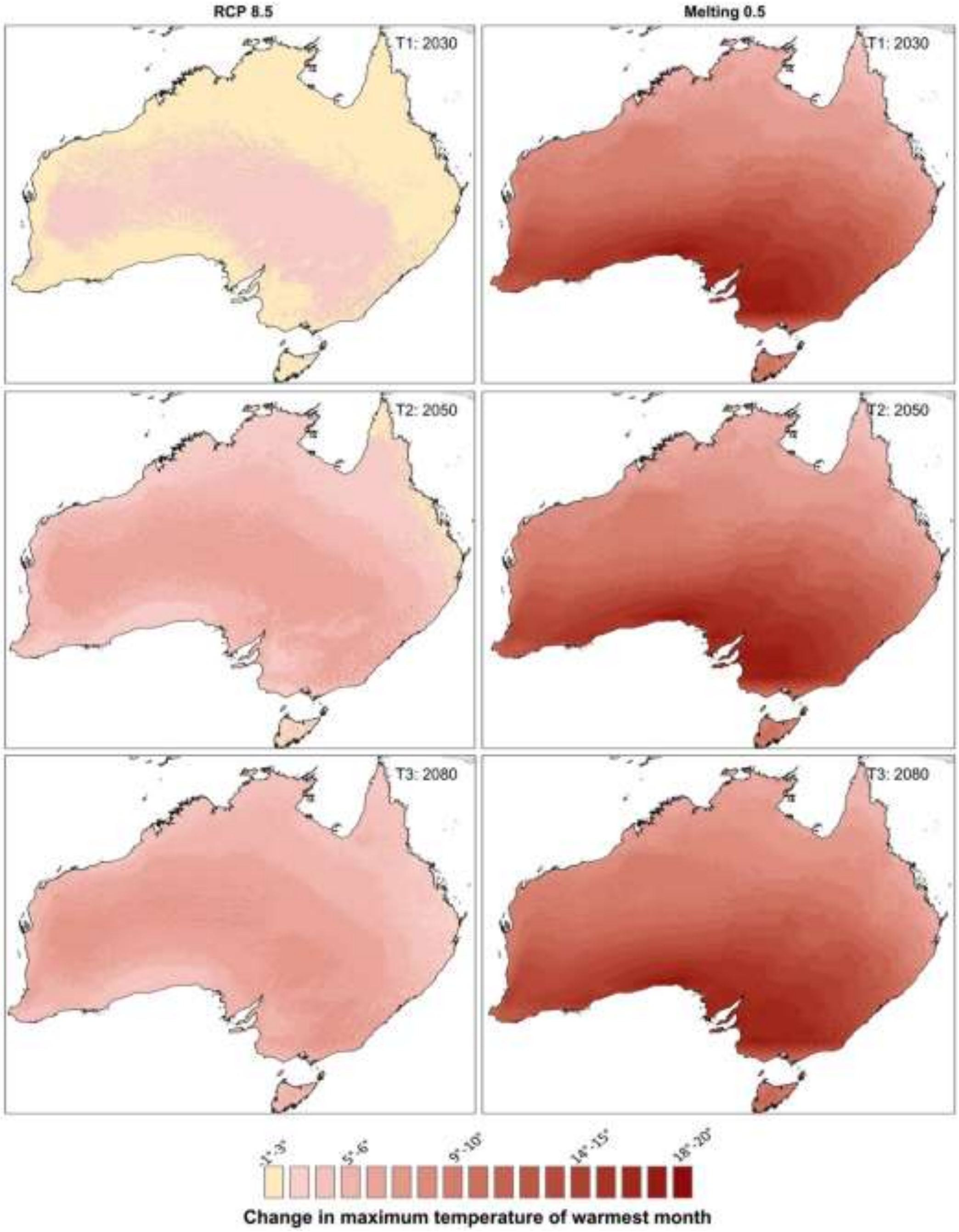

Supplementary Figure 16

Change in maximum temperature of the warmest month (°C). Difference in the maximum temperature of the warmest month between present-day climatic conditions (1970–2000) and future scenarios for Australia at three consecutive time horizons: T1: 2030; T2: 2050; and T3: 2070.

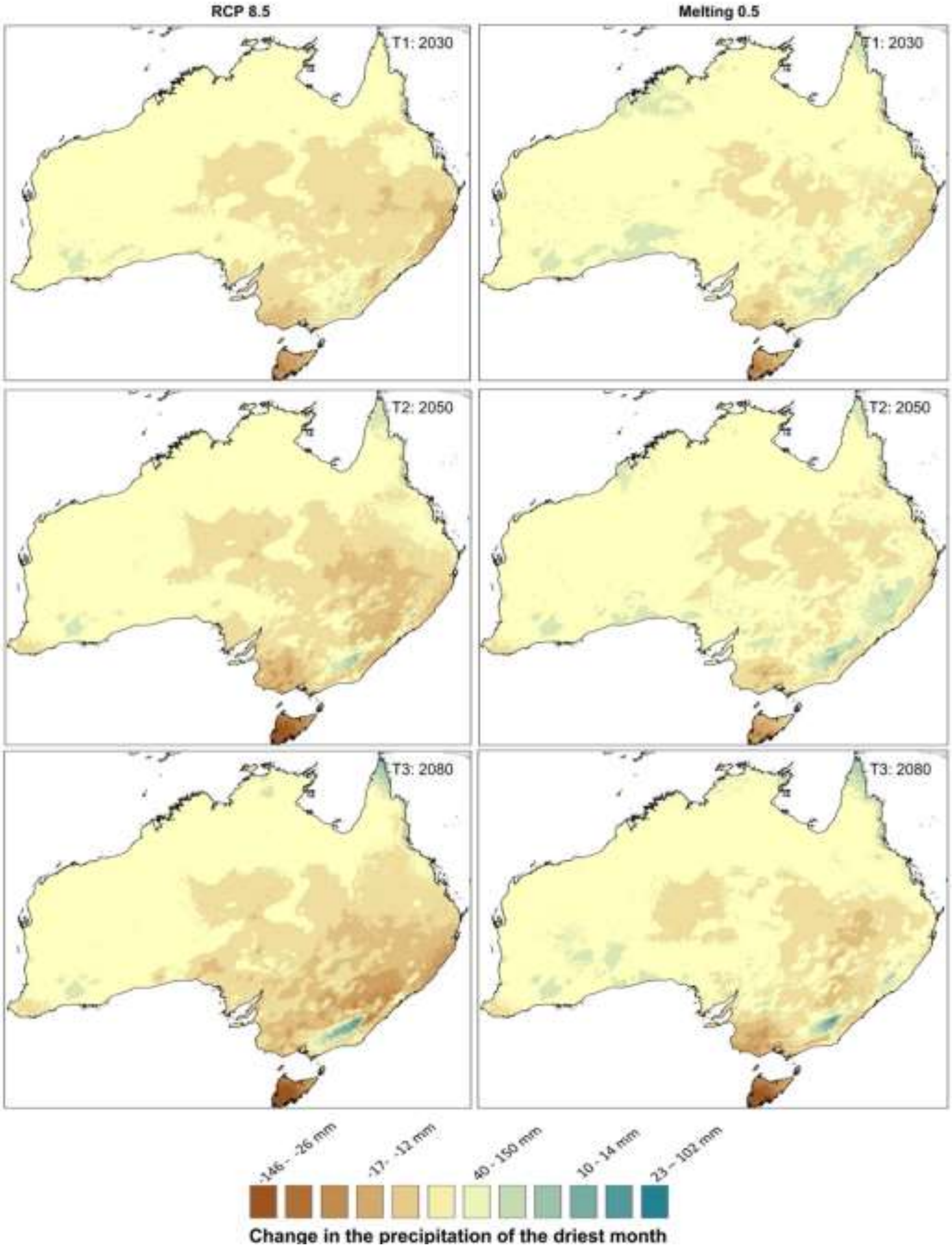

Supplementary Figure 17

Change in precipitation of the driest month (mm). Difference in precipitation of the driest month between present-day climatic conditions (1970–2000) and future scenarios for Australia at three consecutive time horizons: T1: 2030; T2: 2050; and T3: 2070.

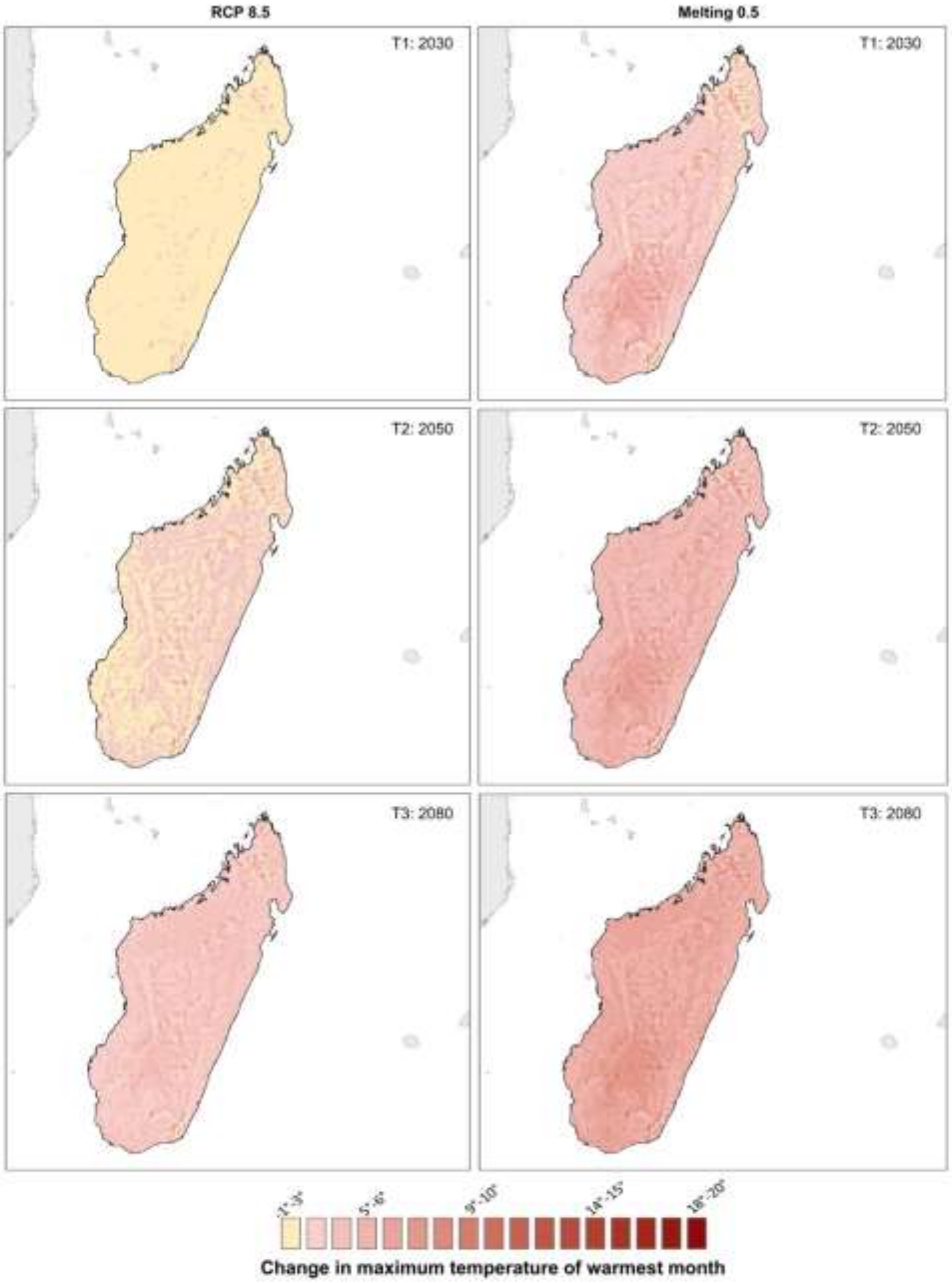

Supplementary Figure 18

Change in maximum temperature of the warmest month (°C). Difference in the maximum temperature of the warmest month between present-day climatic conditions (1970–2000) and future scenarios for Madagascar at three consecutive time horizons: T1: 2030; T2: 2050; and T3: 2070.

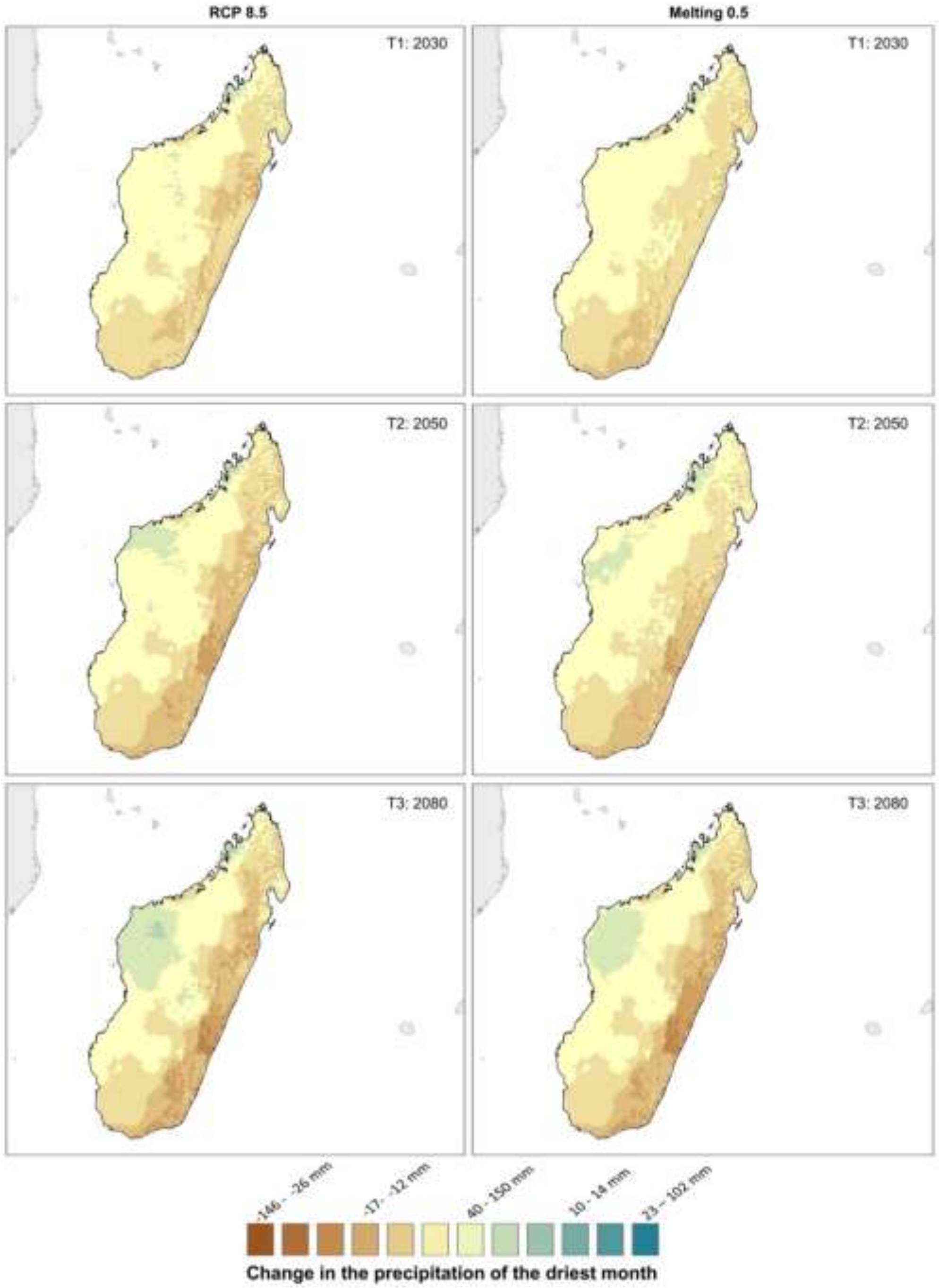

Supplementary Figure 19

Change in precipitation of the driest month (mm). Difference in precipitation of the driest month between present-day climatic conditions (1970–2000) and future scenarios for Madagascar at three consecutive time horizons: T1: 2030; T2: 2050; and T3: 2070.

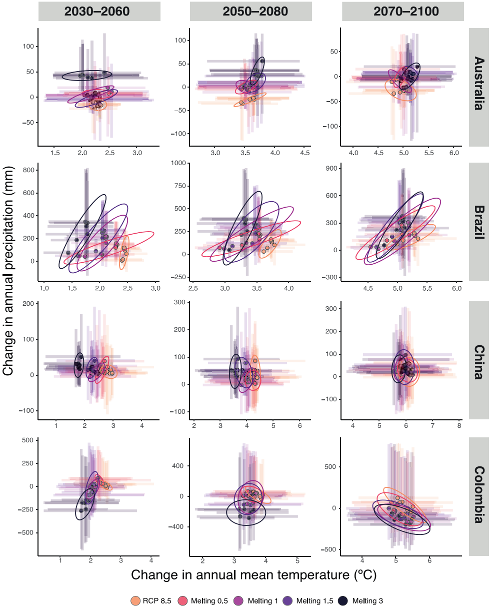

Supplementary Figure 20

Temporal changes in mean annual temperature and precipitation projected across megadiverse countries. Temporal change was estimated as the difference between future climate conditions and present-day climate conditions across four of the twelve countries. Data are groups by country (rows) and temporal horizon (columns). The different taxonomic groups are depicted by circles and cross lines, which depict the median and the corresponding first and third quartiles of species' values within groups, respectively. Colours correspond to the five future climate models. As a visual guide we provide ellipses around data points for every climate model.

24

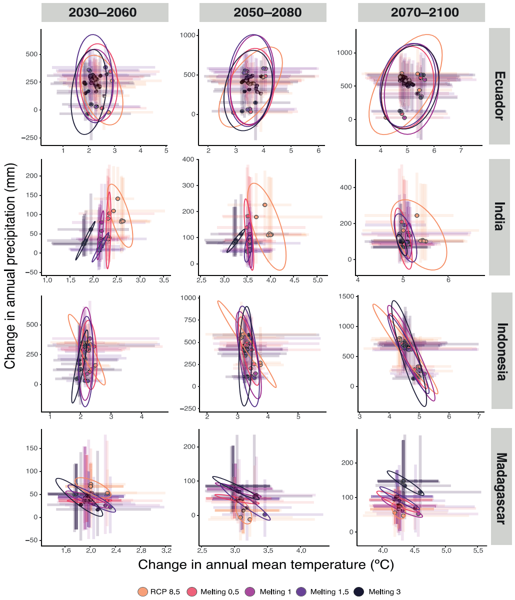

Supplementary Figure 21

Temporal changes in mean annual temperature and precipitation projected across megadiverse countries. Temporal change was estimated as the difference between future climate conditions and present-day climate conditions across four of the twelve countries. Data are groups by country (rows) and temporal horizon (columns). The different taxonomic groups are depicted by circles and cross lines, which depict the median and the corresponding first and third quartiles of species' values within groups, respectively. Colours correspond to the five future climate models. As a visual guide we provide ellipses around data points for every climate model.

25

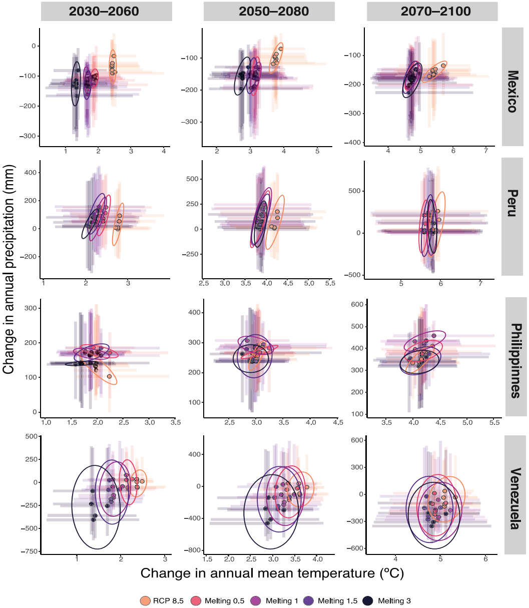

Supplementary Figure 22

Temporal changes in mean annual temperature and precipitation projected across megadiverse countries. Temporal change was estimated as the difference between future climate conditions and present-day climate conditions across four of the twelve countries. Data are groups by country (rows) and temporal horizon (columns). The different taxonomic groups are depicted by circles and cross lines, which depict the median and the corresponding first and third quartiles of species' values within groups, respectively. Colours correspond to the five future climate models. As a visual guide we provide ellipses around data points for every climate model.

26

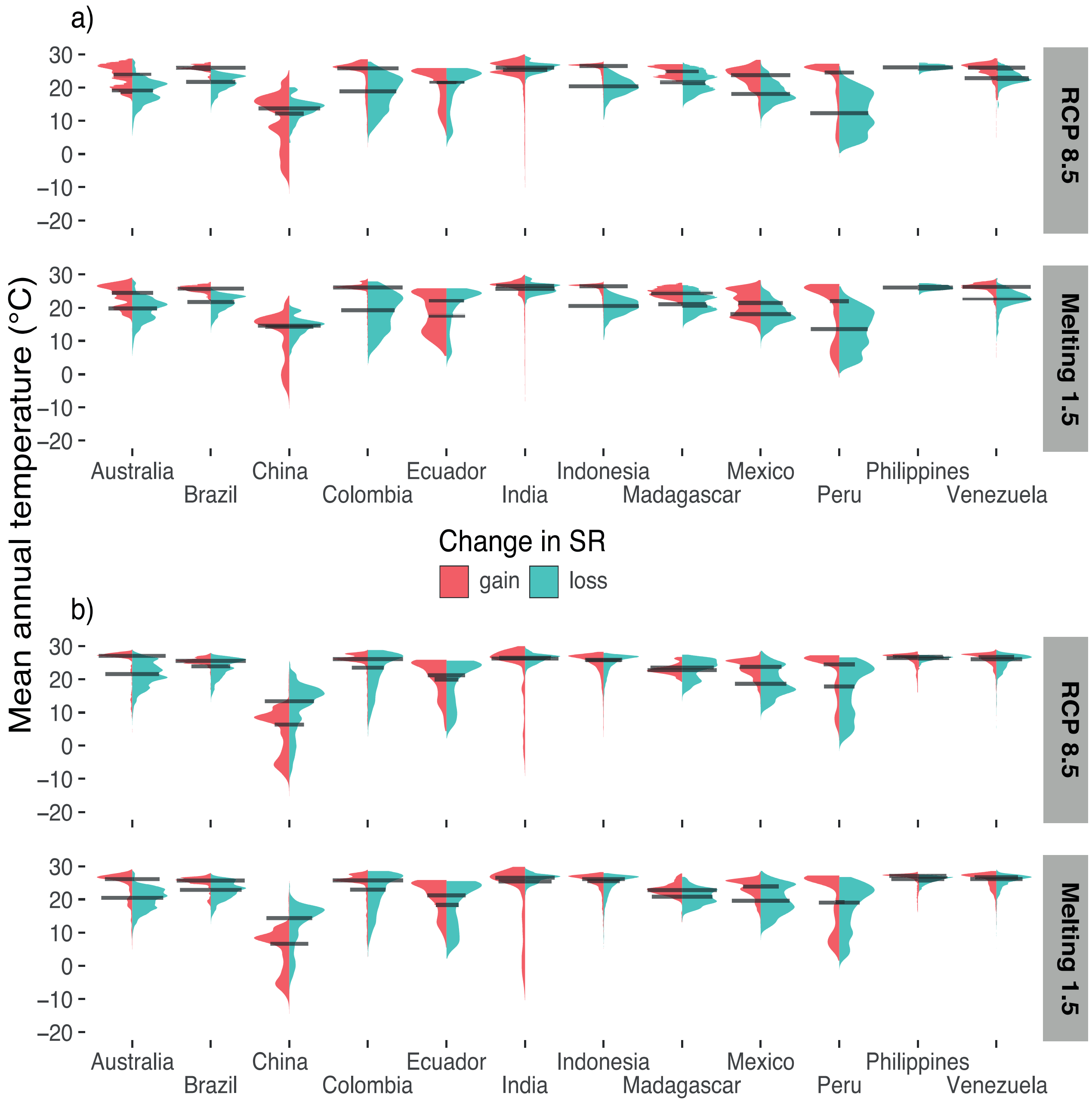

Supplementary Figure 23

Present-day mean annual temperature for areas projected with gain and loss in species richness across twelve megadiverse countries. a–b, estimates are based on species distribution models (SDMs) of (a) vascular plants and (b) tetrapods. Areas of gain and loss represent pixels with differences in species richness above the third quartile and below the first quartile, respectively. The bioclimatic profile for areas is show for 2030–2060 under the RCP 8.5 and Melting 1.5 scenarios.

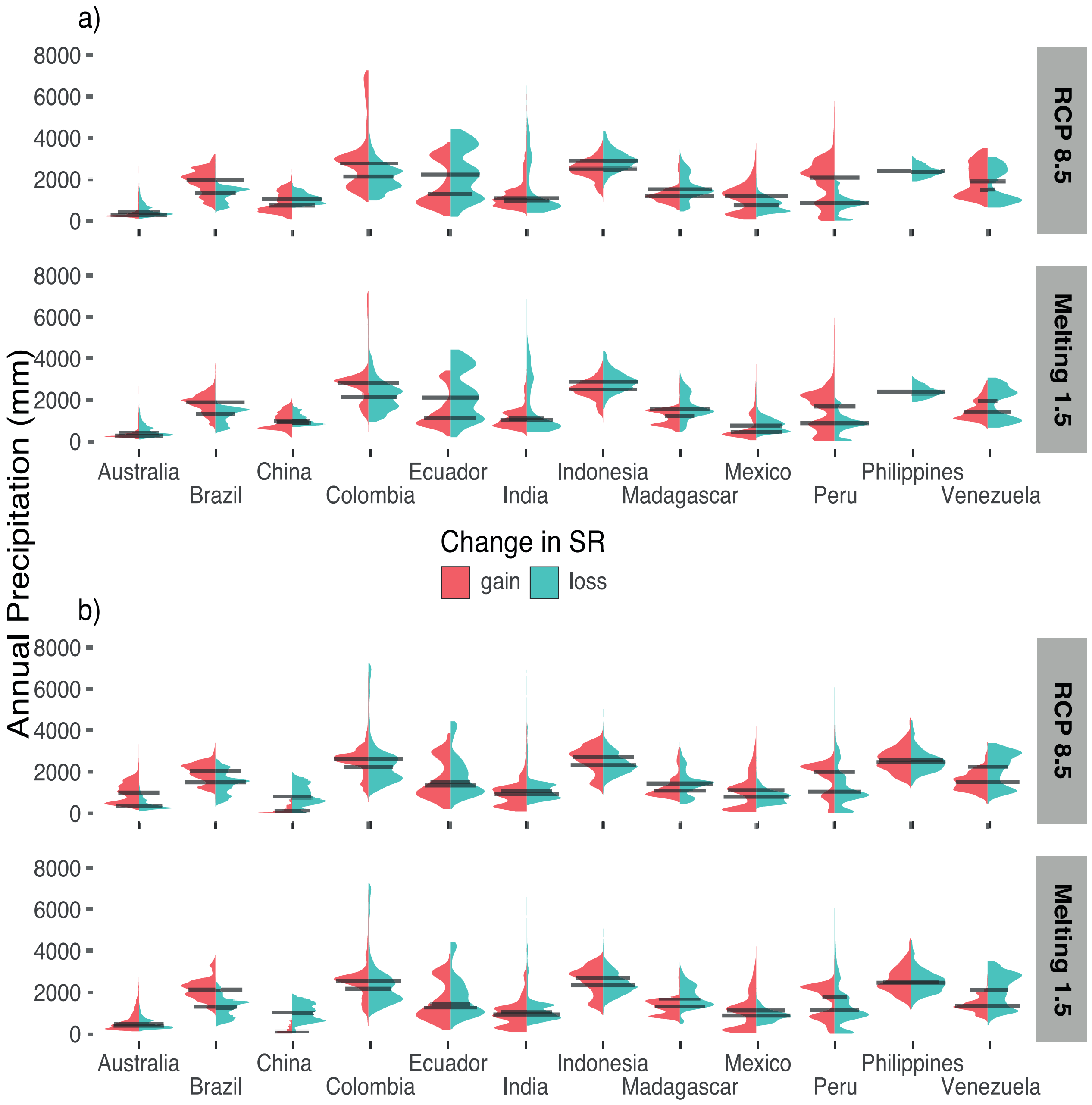

Supplementary Figure 24

Present-day annual precipitation for areas projected with gain and loss in species richness across twelve megadiverse countries. a–b, estimates are based on species distribution models (SDMs) of (a) vascular plants and (b) tetrapods. Areas of gain and loss represent pixels with differences in species richness above the third quartile and below the first quartile, respectively. The bioclimatic profile for areas is show for 2030–2060 under the RCP 8.5 and Melting 1.5 scenarios.

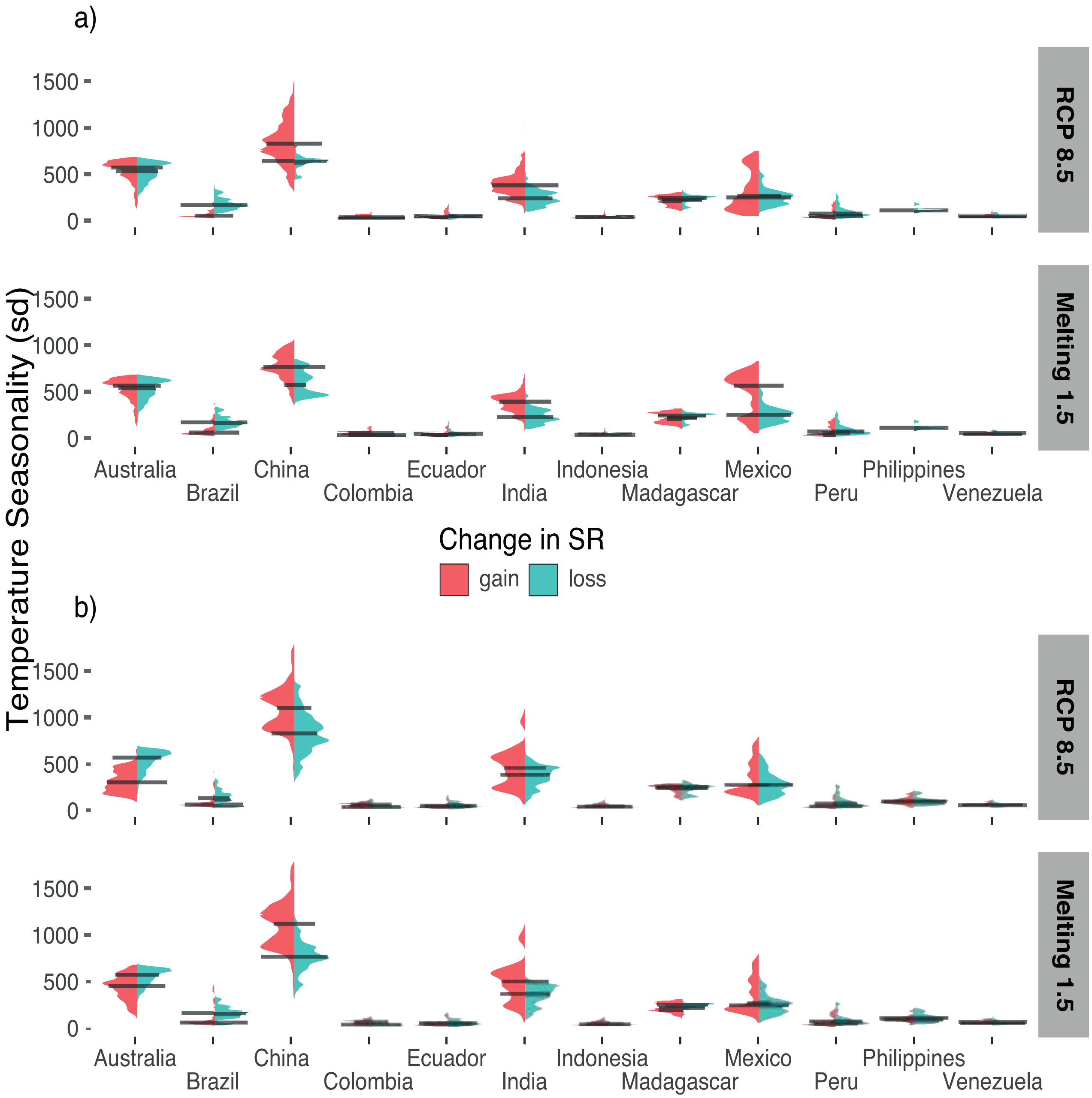

Supplementary Figure 25

Present-day temperature seasonality for areas projected with gain and loss in species richness across twelve megadiverse countries. a–b, estimates are based on species distribution models (SDMs) of (a) vascular plants and (b) tetrapods. Areas of gain and loss represent pixels with differences in species richness above the third quartile and below the first quartile, respectively. The bioclimatic profile for areas is show for 2030–2060 under the RCP 8.5 and Melting 1.5 scenarios.

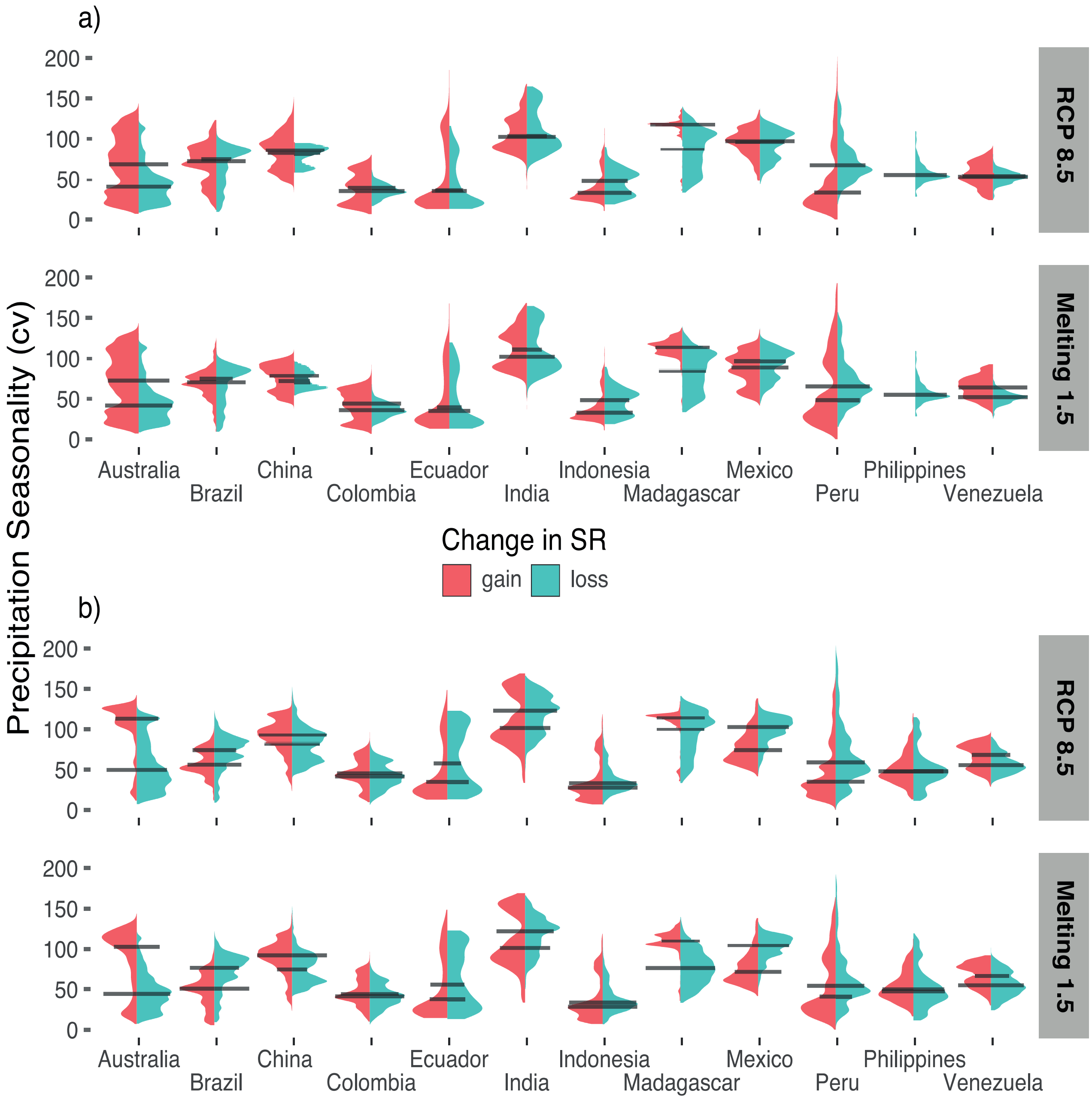

Supplementary Figure 26

Present-day precipitation seasonality for areas projected with gain and loss in species richness across twelve megadiverse countries. a–b, estimates are based on species distribution models (SDMs) of (a) vascular plants and (b) tetrapods. Areas of gain and loss represent pixels with differences in species richness above the third quartile and below the first quartile, respectively. The bioclimatic profile for areas is show for 2030–2060 under the RCP 8.5 and Melting 1.5 scenarios.

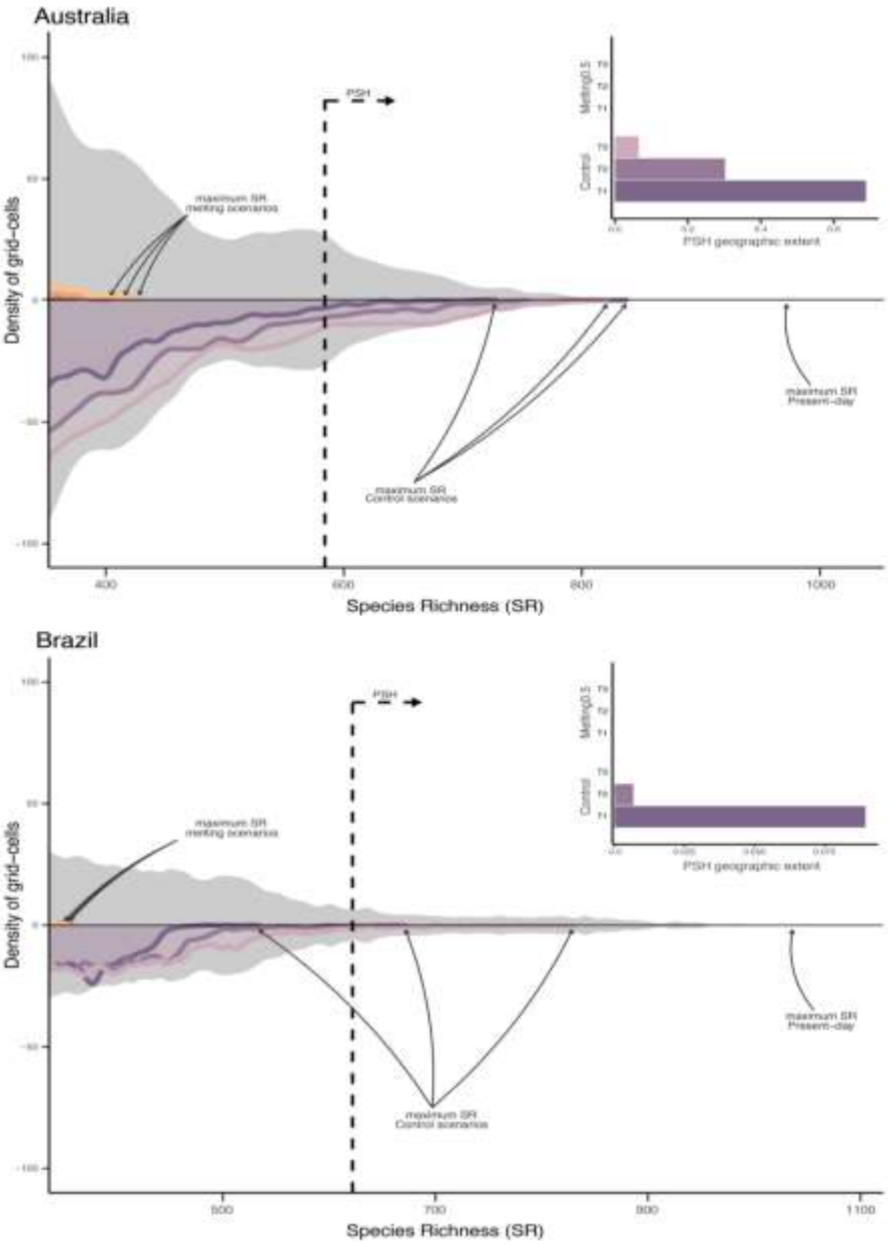

Supplementary Figure 27

Density distribution of species richness (SR) per grid-cell in Australia and Brazil. Species richness estimated under the control scenario RCP 8.5 (bottom half) and the melting scenario Melting 0.5 (top half). Arrows point to the maximum SR values under the different scenarios and the dashed line represents the threshold to define the potential species hotspots (PSH). Inset bar plot depicts the proportional geographical extension of PSH relative to their present-day extension under the two scenarios in different time periods (T1: 2030–2060; T2: 2050–2080; T3: 20670–2100).

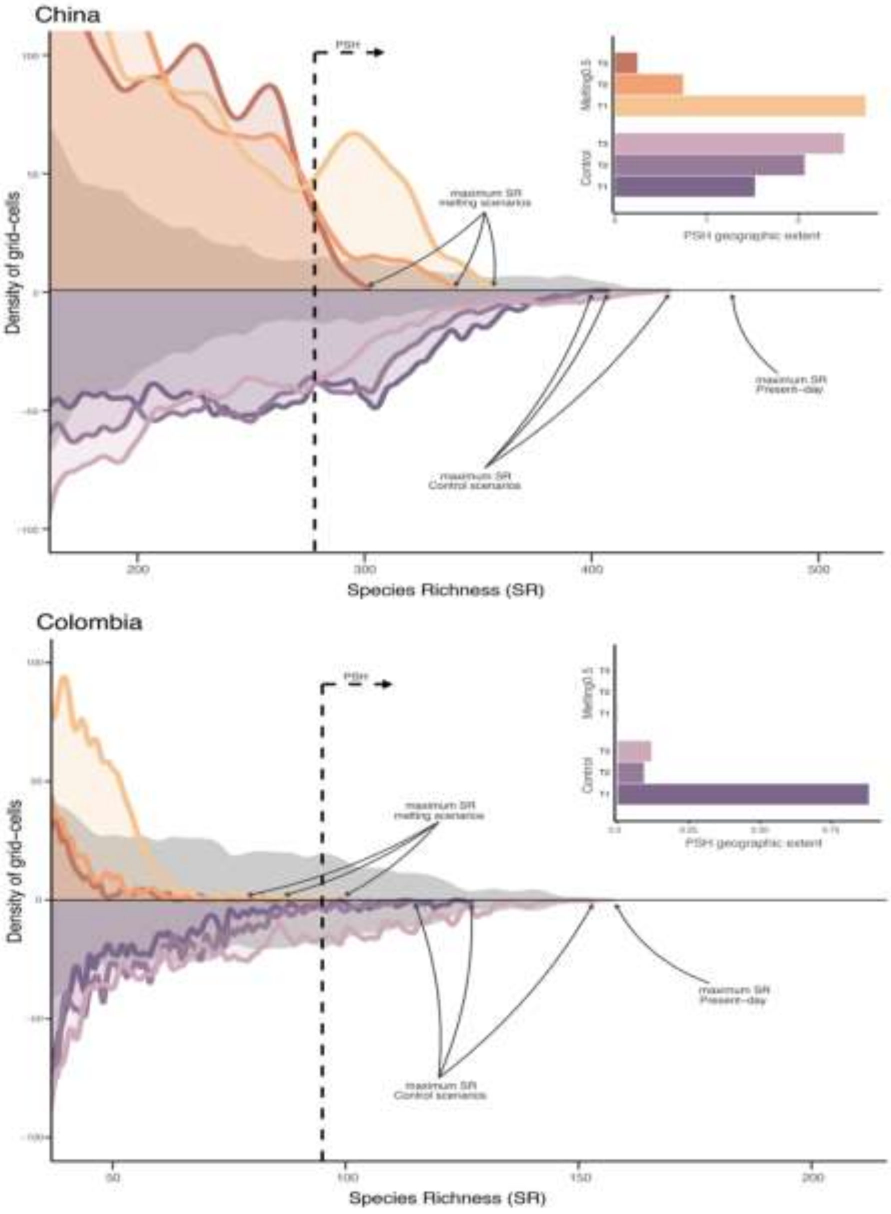

Supplementary Figure 28

Density distribution of species richness (SR) per grid-cell in China and Colombia. Species richness estimated under the control scenario RCP 8.5 (bottom half) and the melting scenario Melting 0.5 (top half). Arrows point to the maximum SR values under the different scenarios and the dashed line represents the threshold to define the potential species hotspots (PSH). Inset bar plot depicts the proportional geographical extension of PSH relative to their present-day extension under the two scenarios in different time periods (T1: 2030–2060; T2: 2050–2080; T3: 20670–2100).

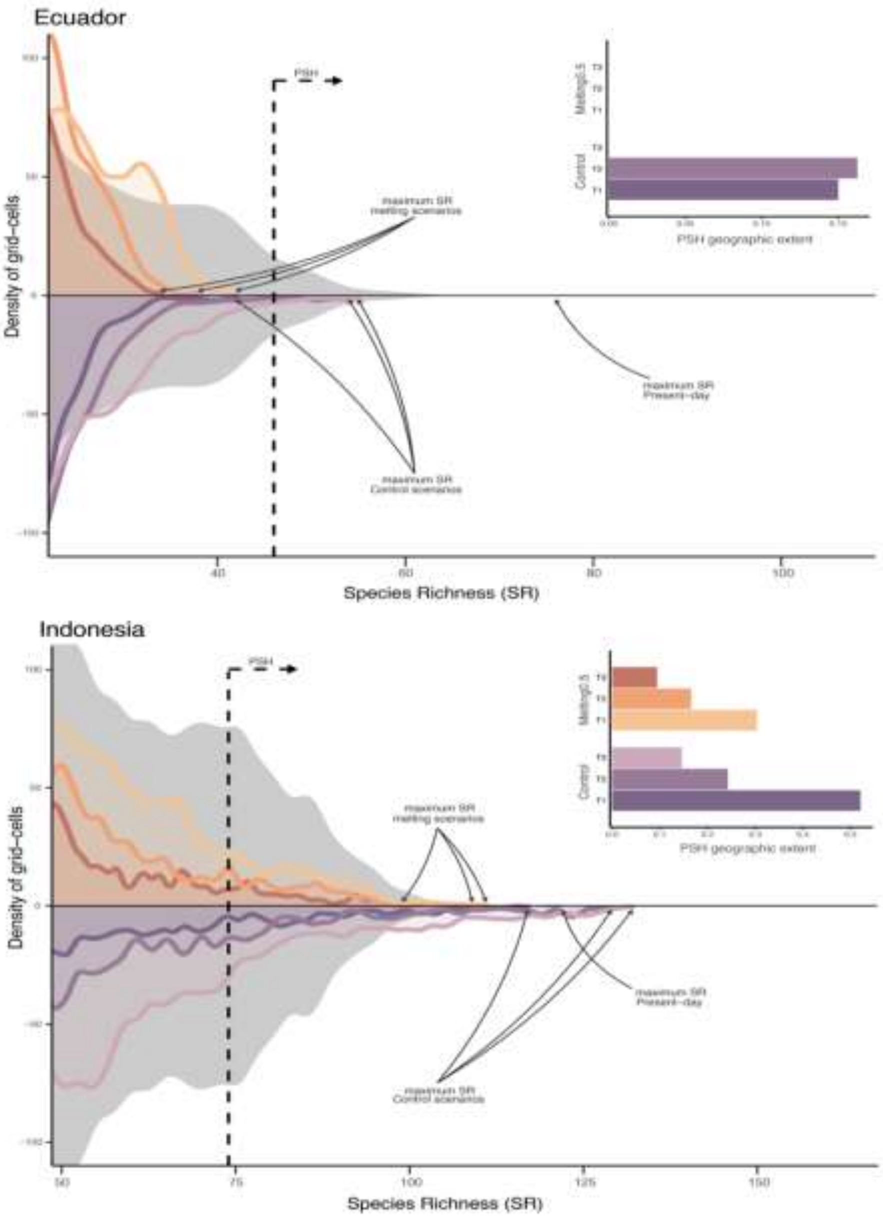

Supplementary Figure 29

Density distribution of species richness (SR) per grid-cell in Ecuador and Indonesia. Species richness estimated under the control scenario RCP 8.5 (bottom half) and the melting scenario Melting 0.5 (top half). Arrows point to the maximum SR values under the different scenarios and the dashed line represents the threshold to define the potential species hotspots (PSH). Inset bar plot depicts the proportional geographical extension of PSH relative to their present-day extension under the two scenarios in different time periods (T1: 2030–2060; T2: 2050–2080; T3: 20670–2100).

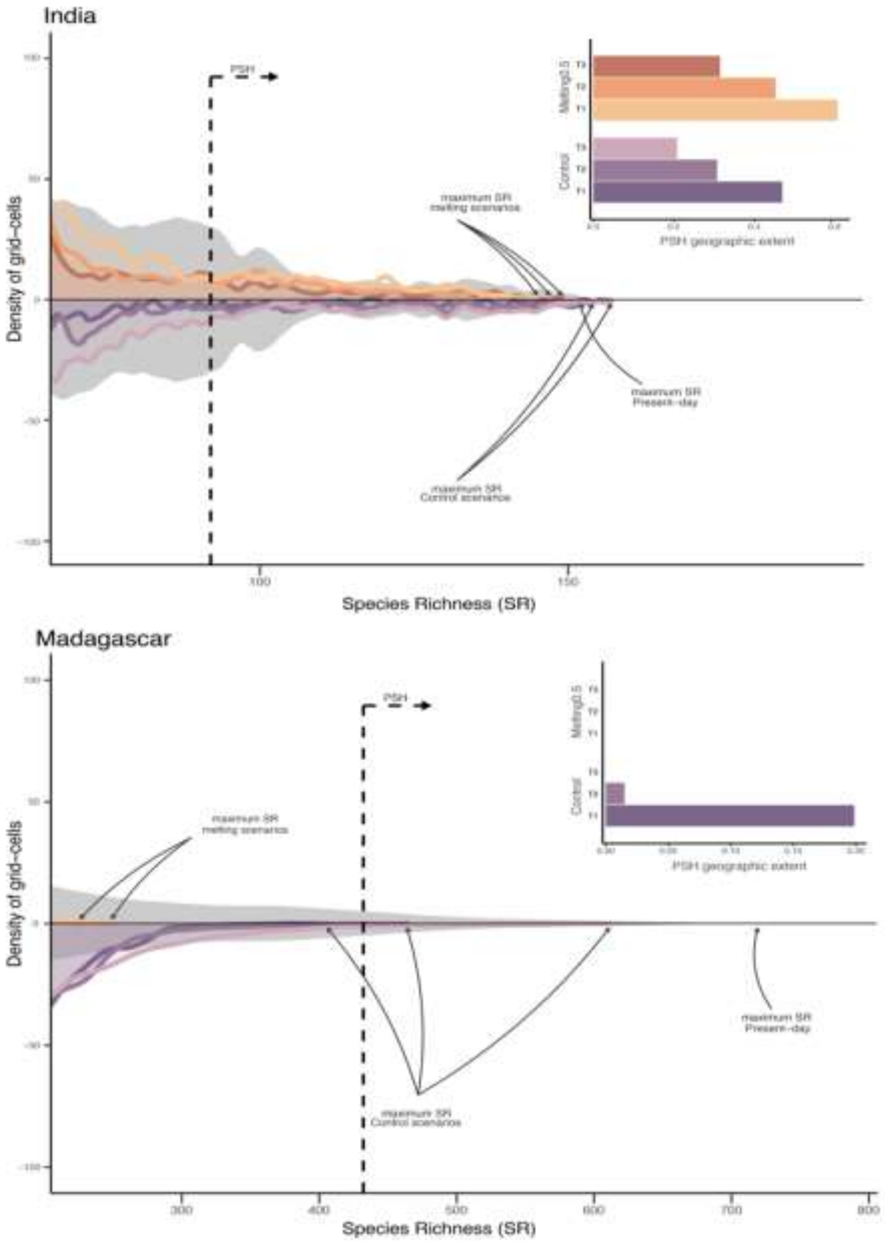

Supplementary Figure 30

Density distribution of species richness (SR) per grid-cell in India and Madagascar. Species richness estimated under the control scenario RCP 8.5 (bottom half) and the melting scenario Melting 0.5 (top half). Arrows point to the maximum SR values under the different scenarios and the dashed line represents the threshold to define the potential species hotspots (PSH). Inset bar plot depicts the proportional geographical extension of PSH relative to their present-day extension under the two scenarios in different time periods (T1: 2030–2060; T2: 2050–2080; T3: 20670–2100).

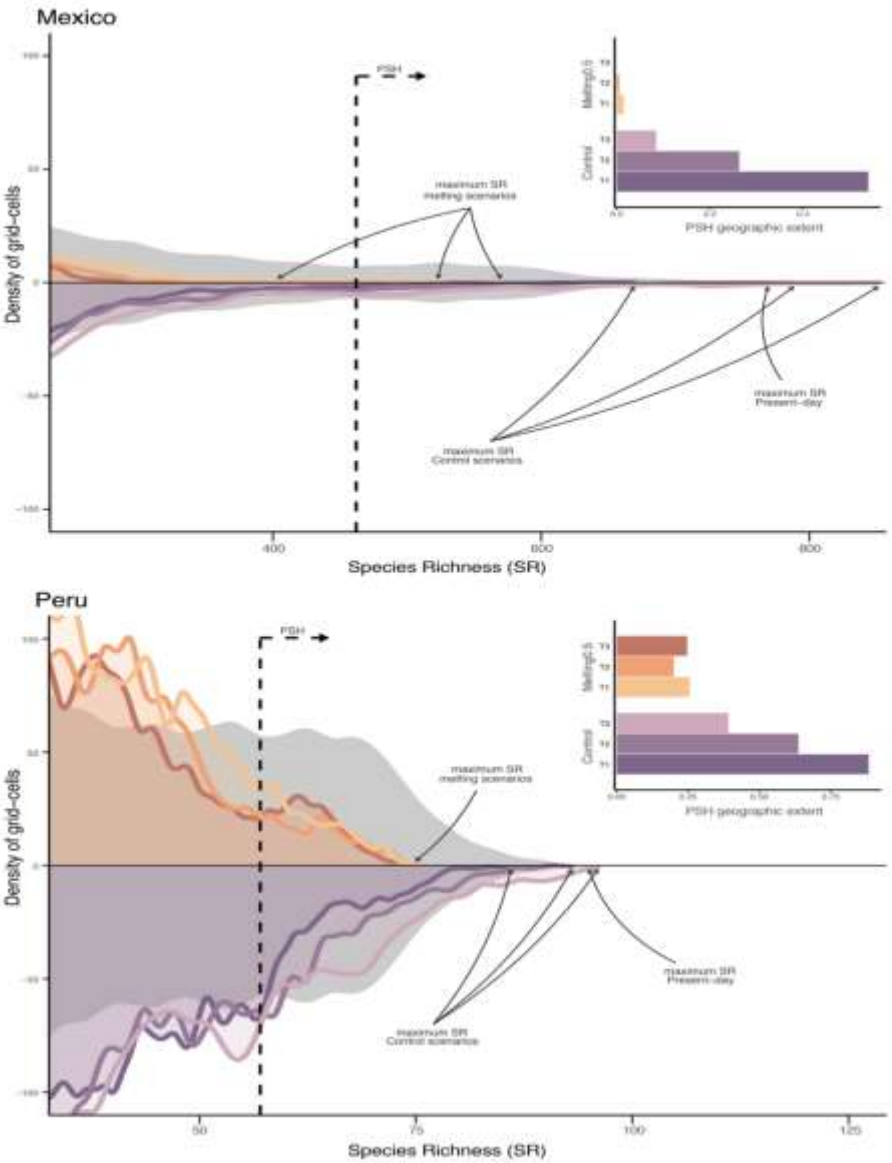

Supplementary Figure 31

Density distribution of species richness (SR) per grid-cell in Mexico and Peru. Species richness estimated under the control scenario RCP 8.5 (bottom half) and the melting scenario Melting 0.5 (top half). Arrows point to the maximum SR values under the different scenarios and the dashed line represents the threshold to define the potential species hotspots (PSH). Inset bar plot depicts the proportional geographical extension of PSH relative to their present-day extension under the two scenarios in different time periods (T1: 2030–2060; T2: 2050–2080; T3: 20670–2100).

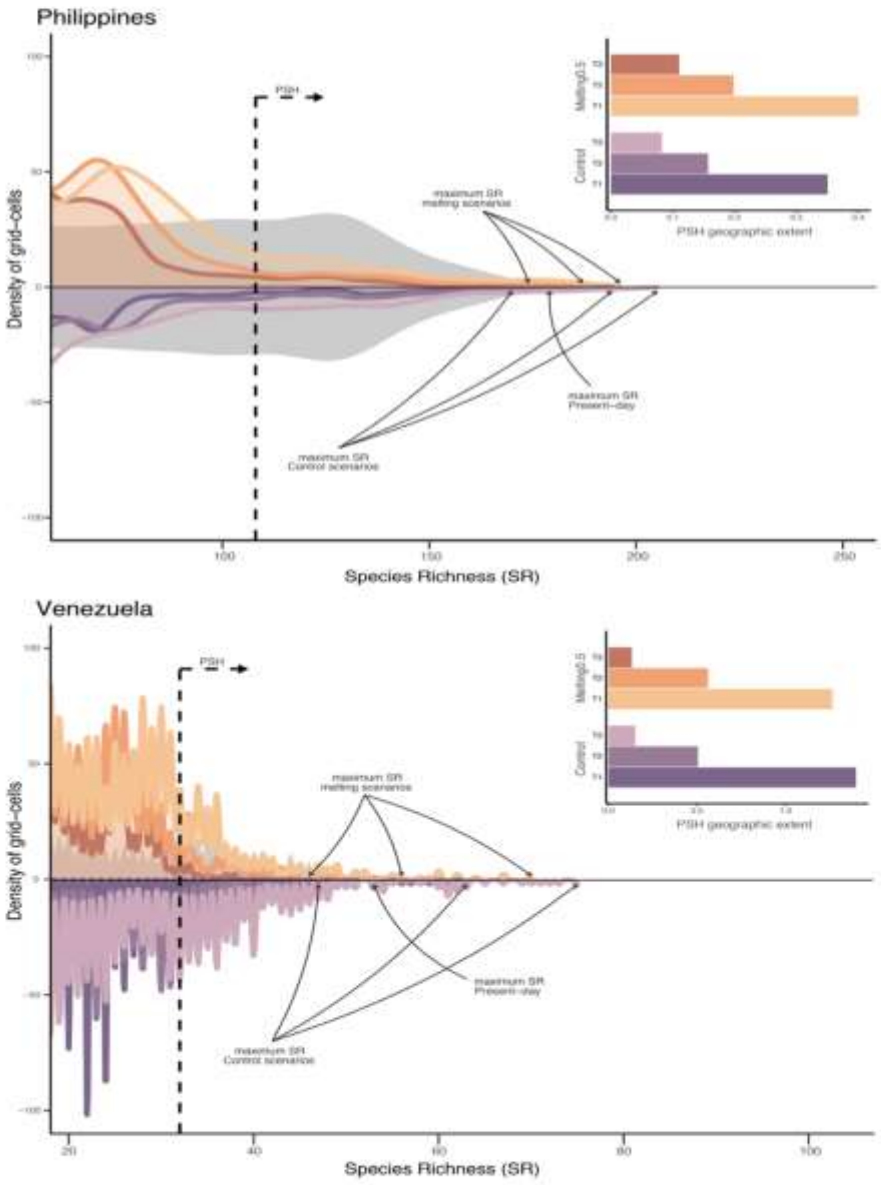

Supplementary Figure 32

Density distribution of species richness (SR) per grid-cell in Philippines and Venezuela. Species richness estimated under the control scenario RCP 8.5 (bottom half) and the melting scenario Melting 0.5 (top half). Arrows point to the maximum SR values under the different scenarios and the dashed line represents the threshold to define the potential species hotspots (PSH). Inset bar plot depicts the proportional geographical extension of PSH relative to their present-day extension under the two scenarios in different time periods (T1: 2030–2060; T2: 2050–2080; T3: 20670–2100).

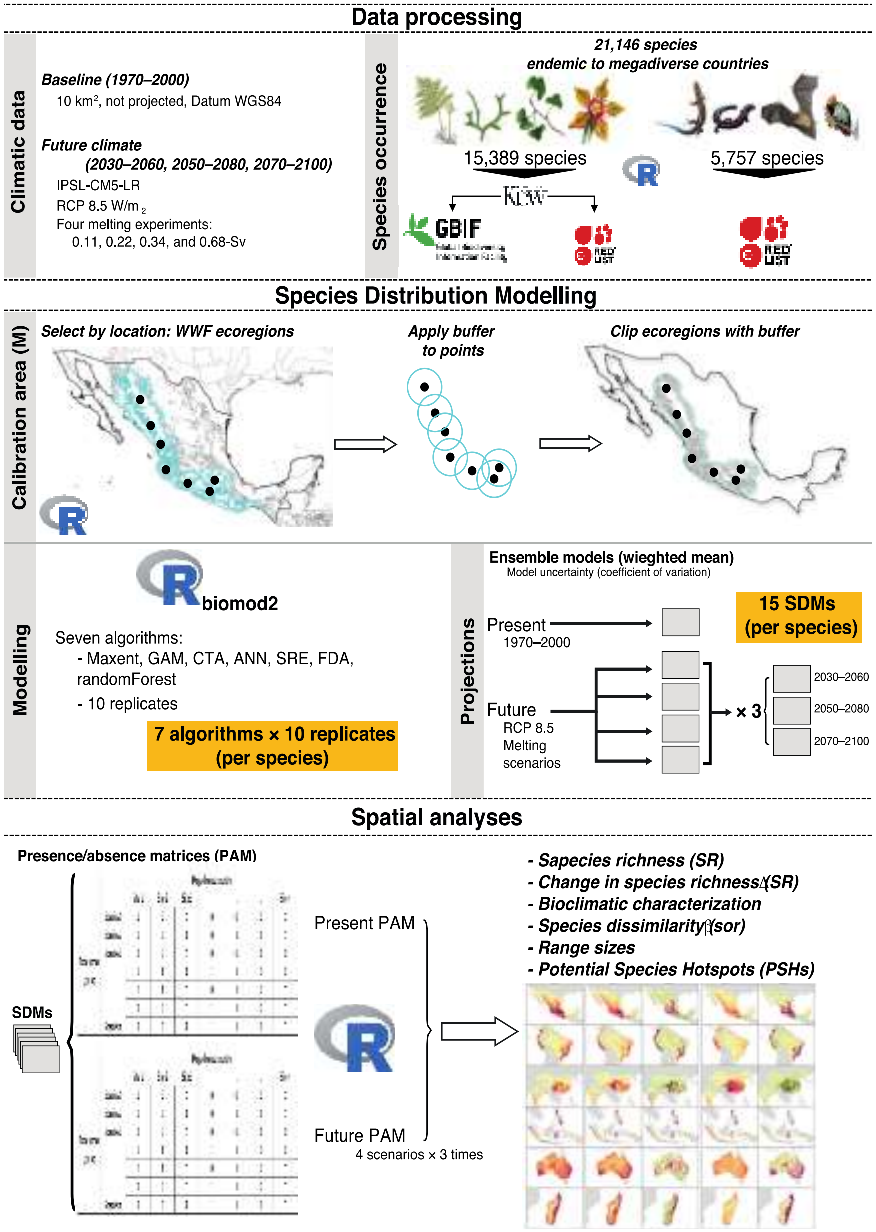

Supplementary Figure 33

Graphical summary of the methodological approach used to model and summarize species distributions. The diagram depicts the main features of the climatic data and species occurrence records we used. We then briefly show how we selected the calibration areas (M) for every species, which is a critical step during species distribution modelling, describe the algorithms used for modelling, and show the underlying logic to projecting the estimated species' climatic profiles across geography and time. Finally, the diagram depicts the construction of presence/absence matrices from the species distribution models and how these matrices are the basis of all the analyses we performed.

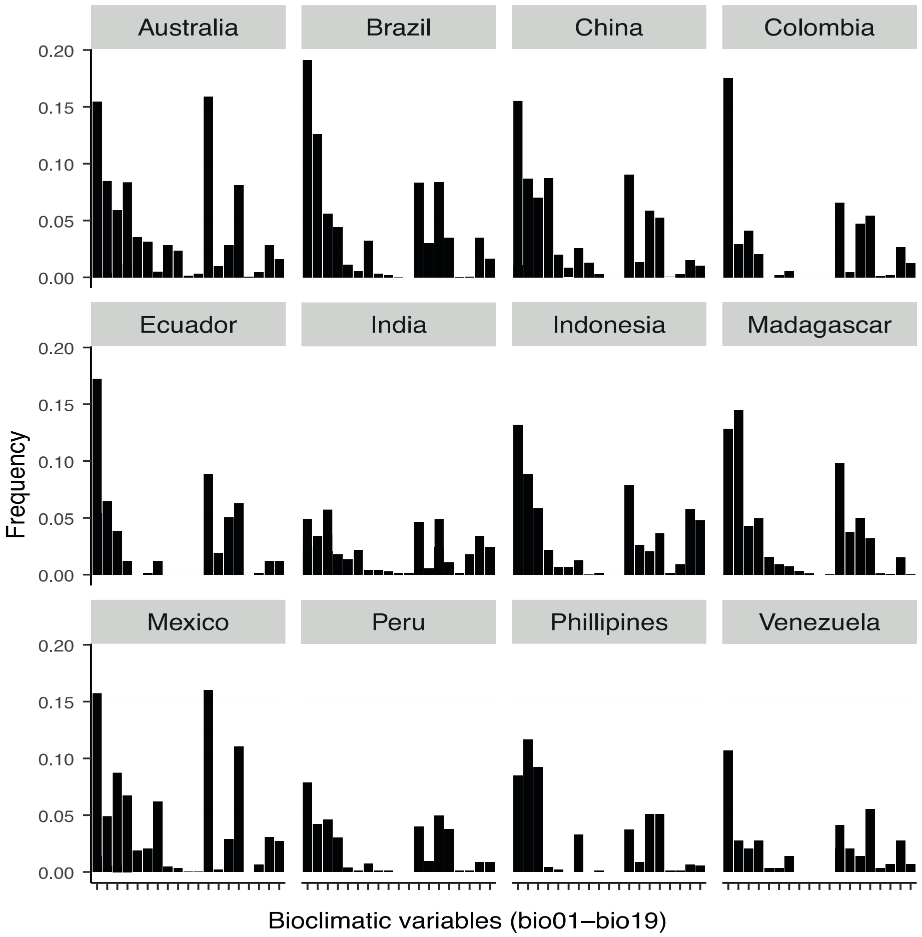

Supplementary Figure 34

Bioclimatic variables with the highest contribution to species distribution models. Frequency of appearance of variables with the highest model contribution across the 21,146 species distribution models grouped by country. Estimates are based on the two variables with the highest contribution for every species model.

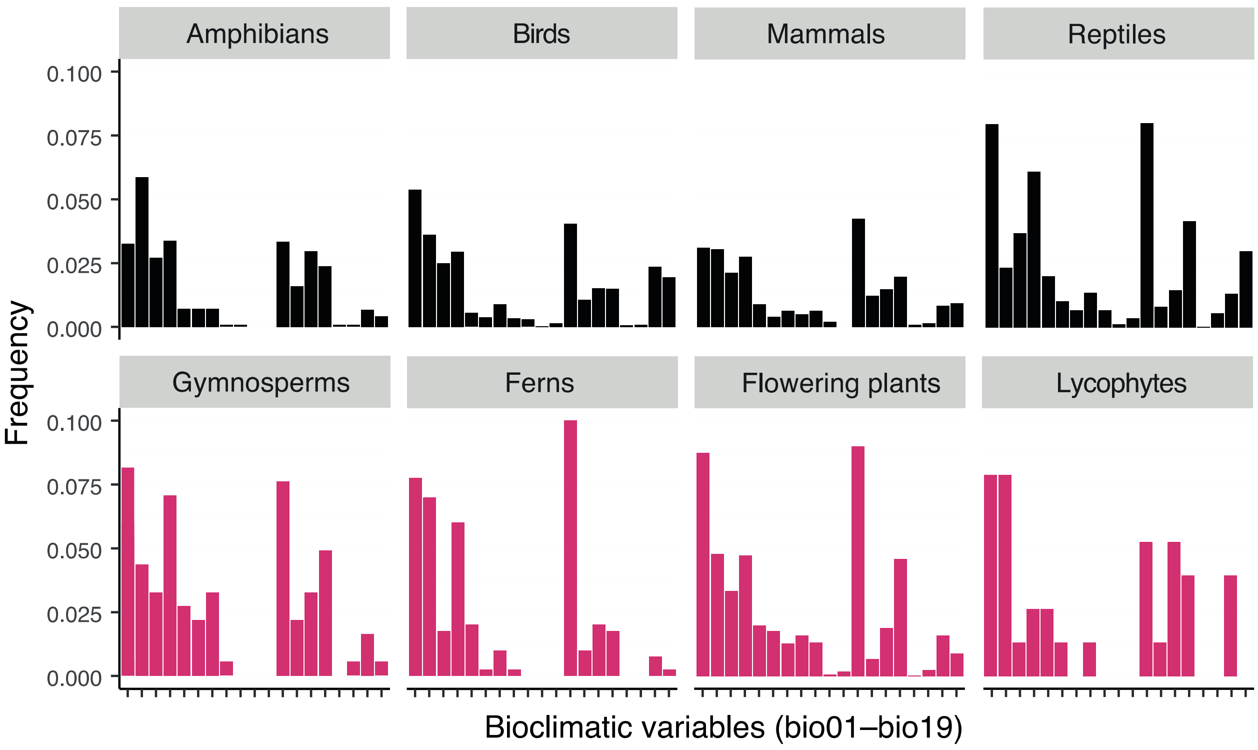

Supplementary Figure 35

Bioclimatic variables with the highest contribution to species distribution models. Frequency of appearance of variables with the highest model contribution across the 21,146 species distribution models grouped by taxonomic group. Estimates are based on the two variables with the highest contribution for every species model.
